# Anatomy and behavioral function of serotonin receptors in *Drosophila melanogaster* larvae

**DOI:** 10.1371/journal.pone.0181865

**Published:** 2017-08-04

**Authors:** Annina Huser, Melanie Eschment, Nazli Güllü, Katharina A. N. Collins, Kathrin Böpple, Lyubov Pankevych, Emilia Rolsing, Andreas S. Thum

**Affiliations:** 1 Department of Biology, University of Konstanz, Konstanz, Germany; 2 Zukunftskolleg, University of Konstanz, Konstanz, Germany; 3 Department of Genetics, University of Leipzig, Leipzig, Germany; Tohoku University, JAPAN

## Abstract

The biogenic amine serotonin (5-HT) is an important neuroactive molecule in the central nervous system of the majority of animal phyla. 5-HT binds to specific G protein-coupled and ligand-gated ion receptors to regulate particular aspects of animal behavior. In *Drosophila*, as in many other insects this includes the regulation of locomotion and feeding. Due to its genetic amenability and neuronal simplicity the *Drosophila* larva has turned into a useful model for studying the anatomical and molecular basis of chemosensory behaviors. This is particularly true for the olfactory system, which is mostly described down to the synaptic level over the first three orders of neuronal information processing. Here we focus on the 5-HT receptor system of the *Drosophila* larva. In a bipartite approach consisting of anatomical and behavioral experiments we describe the distribution and the implications of individual 5-HT receptors on naïve and acquired chemosensory behaviors. Our data suggest that *5-HT*_*1A*_, *5-HT*_*1B*_, and *5-HT*_*7*_ are dispensable for larval naïve olfactory and gustatory choice behaviors as well as for appetitive and aversive associative olfactory learning and memory. In contrast, we show that 5-HT/*5-HT*_*2A*_ signaling throughout development, but not as an acute neuronal function, affects associative olfactory learning and memory using high salt concentration as a negative unconditioned stimulus. These findings describe for the first time an involvement of 5-HT signaling in learning and memory in *Drosophila* larvae. In the longer run these results may uncover developmental, 5-HT dependent principles related to reinforcement processing possibly shared with adult *Drosophila* and other insects.

## Introduction

The biogenic amine serotonin (5-HT) exerts an essential role in a wide range of insect behaviors by its action as neurotransmitter, neuromodulator, and/or neurohormone (reviewed by [1]). Accordingly, for the adult fruit fly *Drosophila melanogaster* it was shown that 5-HT signaling is involved in chemosensation [2, 3], aggression [4, 5], mating [6], feeding [7, 8], and locomotion [7, 9].

As in vertebrates, 5-HT acts as natural ligand for a group of G protein-coupled receptors and ligand-gated ion channels found in the central and peripheral nervous systems [10–12]. 5-HT receptors mediate both excitatory and inhibitory function. In the *Drosophila* genome five different 5-HT G protein-coupled receptors have been previously identified, called *5-HT*_*1A*_, *5-HT*_*1B*_, *5-HT*_*2A*_, *5-HT*_*2B*_, and *5-HT*_*7*_ [13–18]. 5-HT receptor expression was found in distinct adult fly brain regions including the mushroom bodies (MB), central complex, optic lobes and antennal lobes (AL) [19–24]. Consequently, differential 5-HT receptor requirement was reported for aggression (*5-HT*_*1A*_ and *5-HT*_*2A*_), sleep (*5-HT*_*1A*_), feeding (*5-HT*_*1B*_ and *5-HT*_*2A*_), circadian entrainment and behavior (*5-HT*_*1B*_ and *5-HT*_*2A*_), and courtship and mating (*5-HT*_*7*_) [8, 19–21, 23, 25, 26]. Moreover, 5-HT/5-HT receptor signaling was shown to be required for learning and memory as *5-HT*_*1A*_, *5-HT*_*2A*_, and *5-HT*_*7*_ were reported to be involved in short and long term association [27]. Additionally, anesthesia-resistant memory formation was supposed to be mediated through *5-HT*_*1A*_ receptors expressed in the mushroom body [28].

At the larval stage only a few studies have addressed the anatomical organization and functional relevance of 5-HT receptors. It was described that putative *5-HT*_*1A*_, *5-HT*_*1B*_, *5-HT*_*2A*_, and *5-HT*_*7*_ positive cells can be found in the larval central nervous system (CNS) [19, 21, 23, 29]. This allows 5-HT to specifically regulate certain aspects of larval physiology and behavior. In detail, distinct larval 5-HT receptor function was reported for locomotion (*5-HT*_*1A*_, *5-HT*_*1B*_, *5-HT*_*2A*_, *5-HT*_*2B*_, and *5-HT*_*7*_), reduced light avoidance (*5-HT*_*1A*_), juvenile-to-adult transition (*5-HT*_*7*_), feeding (*5-HT*_*2A*_), and modulation of heart rate frequency (*5-HT*_*2A*_ and *5-HT*_*2B*_) [13, 30–34].

However, these studies often focus on a single type of 5-HT receptor and thus miss a comprehensive side-by-side investigation of each receptor on the anatomical and behavioral level. Accordingly, one emphasis of this study was to investigate the organization of several different 5-HT receptors within the larval brain in parallel. To this end we used the *Gal4*-UAS system that allows for reproducible expression of effector genes in defined subsets of potential 5-HT receptor cells [35–37]. Based on the limitation of available *Gal4* lines we focused our analysis on four of the five 5-HT receptors: *5-HT*_*1A*_*-Gal4*, *5-HT*_*1B*_*-Gal4*, *5-HT*_*2A*_*-Gal4* (also often called *5-HT*_*2Dro*_*-Gal4* [21]), and *5-HT*_*7*_*-Gal4*. Please note that *5-HT*_*2B*_ was only recently identified [13] and it is not part of this study. Thereby, we provide an initial analysis of larval 5-HT receptor system based on genetic tools that are state of the art and widely used in the field. We want to emphasize that it is not clear for all 5-HT receptor *Gal4* lines if and how reliable the *Gal4* expression reflects the endogenous receptor gene expression patterns. Due to technical limitations antibody and in-situ information is almost completely unavailable. Consequently, in the longer run, more sophisticated genetic tools have to be established and co-localization studies have to be performed.

Here, we applied a two-part approach by analyzing the expression patterns and the behavioral function of potential 5-HT receptor cells in *Drosophila* larvae. The behavioral assays include measurements for naïve olfactory and gustatory preferences performed via simple choice tests on agarose filled test plates [38]. In addition, a more advanced design allows to study associative olfactory learning and memory [39–47]. Presenting an odor (the conditioned stimulus (CS)) simultaneously with an aversive unconditioned stimulus (US) induces experience dependent avoidance of the CS. Conversely, if the same CS is paired with an appetitive US, animals can be trained to develop a preference for the CS. Thus, depending on previous experience, the same odor can trigger either avoidance or attraction [41]. Taken together, a comprehensive set of behavioral assays to analyze larval chemosensory behavior is at hand that allows investigating simple choice behavior and associative olfactory learning and memory in *Drosophila* larvae.

In an earlier study, we could show that 5-HT positive cells are *per se* neither necessary for naïve gustatory, olfactory, or light preferences nor for associative olfactory learning and memory (using fructose and electric shock as positive and negative reinforcers, respectively) [48]. However, this does not exclude a specific contribution of distinct 5-HT receptors for chemosensory behaviors. Different 5-HT receptors act antagonistically. *5-HT*_*7*_ was shown to activate adenylyl cyclases and increases cAMP levels, whereas *5-HT*_*1A*_ and *5-HT*_*1B*_ receptors inhibit cAMP production [14, 15, 18]. Thus, ablation of the entire 5-HT system can affect both assisting and inhibitory functions of antagonistic 5-HT receptors at the same time. We have therefore expanded our analysis on the role of 5-HT signaling on larval chemosensory behavior by focusing on distinct 5-HT receptor functions.

## Materials and methods

### Flies

Flies were maintained on standard *Drosophila* medium at 25°C or 19°C under 12h light /dark conditions. All 5-HT receptor specific lines, *5-HT*_*1A*_*-Gal4* [29], *5-HT*_*1B*_*-Gal4* [49] (Bloomington Stock Center no. 24240), *5-HT*_*2A*_*-Gal4* [21, 50] (Bloomington Stock Center no. 19367), *5-HT*_*7*_*-Gal4* [23], were kindly provided by Charles Nichols. Note that the *5-HT*_*2A*_*-Gal4* construct is an enhancer trap piggyBac construct in the *5-HT*_*2A*_ locus that reduces its expression by nearly 90% [21]. *TRH-GAL4* [78] was kindly provided by Serge Birman. For behavioral experiments, wild type *Canton-S* (*WT CS*) flies and the effector lines UAS-*hid*,*rpr* [51, 52] and UAS-*shi*^*ts*^ [53] (Bloomington Stock Center no. 44222) were used. *w*^*1118*^ flies (kindly provided by Martin Heisenberg) were crossed with UAS- and *Gal4* lines to obtain heterozygous controls. To visualize the *Gal4* expression pattern, we used UAS-*mCD8*::*GFP* [54] (kindly provided by Hiromu Tanimoto) and UAS-myr::tomato [55, 56] (Bloomington Stock Center no. 32221). In all cases five to six day old feeding third instar larvae were used.

### Immunostaining

**Fig 1:**
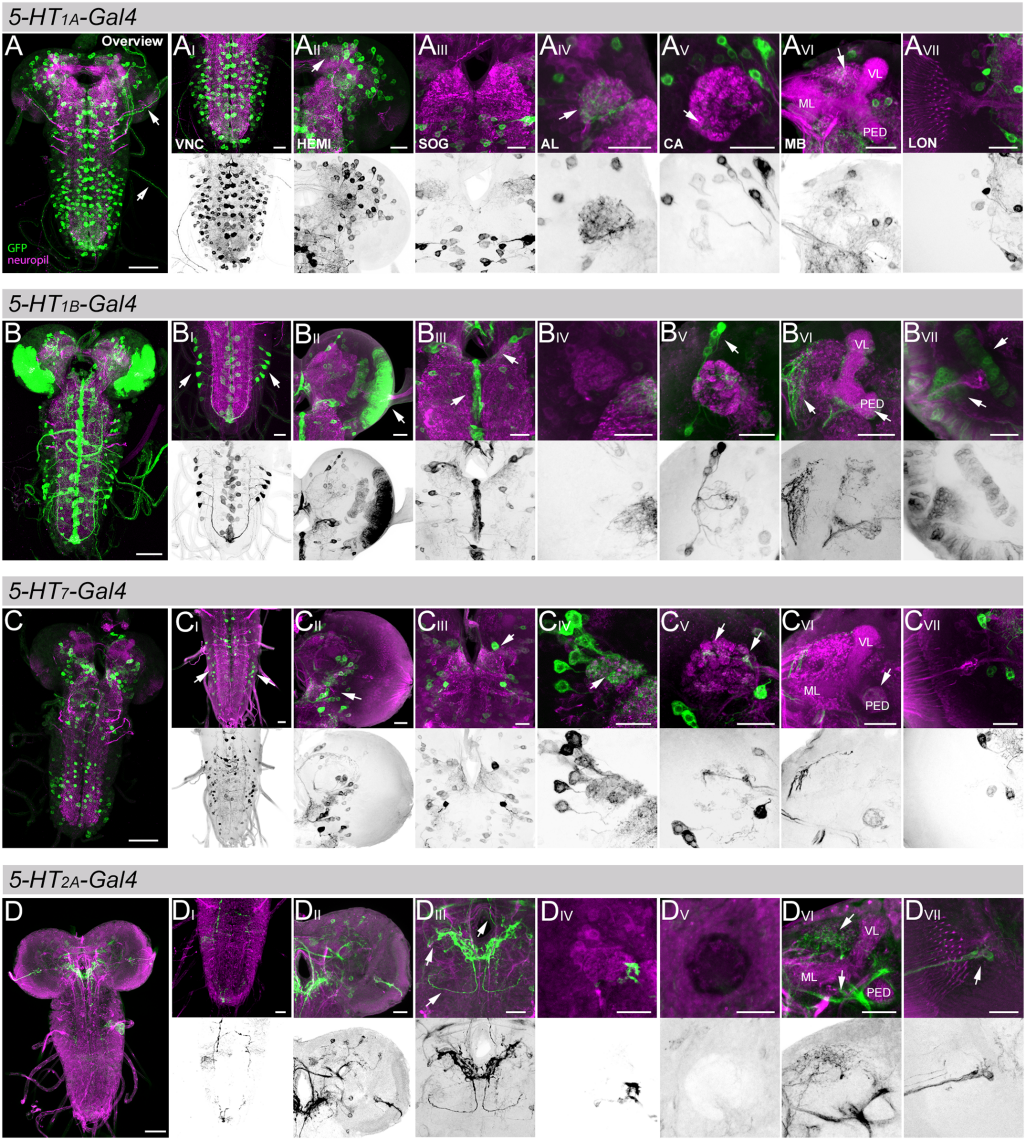
*Gal4* expression patterns of four potential 5-HT receptor lines. *5-HT*_*1A*_-, *5-HT*_*1B*_-, *5-HT*_*7*_-, and *5-HT*_*2A*_*-Gal4* positive cells are shown in A, B, C, and D, respectively. (A-C) *Gal4* lines were crossed with UAS-*mCD8*::*GFP* to analyze their expression patterns (green; anti-GFP staining) in addition to reference labeling of the central nervous system (CNS) (magenta; anti-ChAT/anti-FasII double-staining). In (D) *5-HT*_*2A*_*-Gal4* was crossed with UAS-*myr*::*tomato* to visualize its expression pattern (green; anti-dsRed staining) within the larval CNS (magenta; anti-ChAT/anti-FasII double-staining). For all four lines the first column shows a frontal view onto a z-projection of the entire CNS. In addition, for each line representative z-projections of close-ups of the ventral nerve cord (VNC), one hemisphere (HEMI), the suboesophageal ganglion (SOG), one antennal lobe (AL), the calyx (CA) of the mushroom body (MB), the lobes of the MB, and the larval optic neuropil (LON) are shown from left to right. Below each close-up only the GFP channel is shown as an inverted black and white image to visualize innervation patterns with higher contrast and no neuropil background. White arrows highlight aspects of the expression patterns that are further described in the results. Additional abbrevations: VL vertical lobe, ML medial lobe, PED peduncle; Scale bars: 50 μm (in A, B, C, D) and 20 μm (in all other panels).

Experiments were performed as described before [57, 58]. Third instar larvae were put on ice and dissected in phosphate buffered saline (PBS). Brains were fixed in 3.6% formaldehyde (Merck, Darmstadt) in PBS for 30 min. After washing with PBT (PBS with 3% Triton-X 100, Sigma Aldrich, St. Louis, MO), brains were blocked with 5% normal goat serum (Vector Laboratories, Burlingame, CA) in PBT for one to two hours and then incubated for two days with first antibodies at 4°C. Before applying the secondary antibodies for one or two days at 4°C, brains were washed with PBT. Finally, brains were again washed, mounted in Vectashield (Vector Laboratories) between two cover slips and stored at 4°C in darkness. This protocol was used for data presented in Figs 1A, 1A_I_, 1A_II_, 1B, 1B_I_, 1B_VI_, 1C, 1C_IV_ and 2.

**Fig 2:**
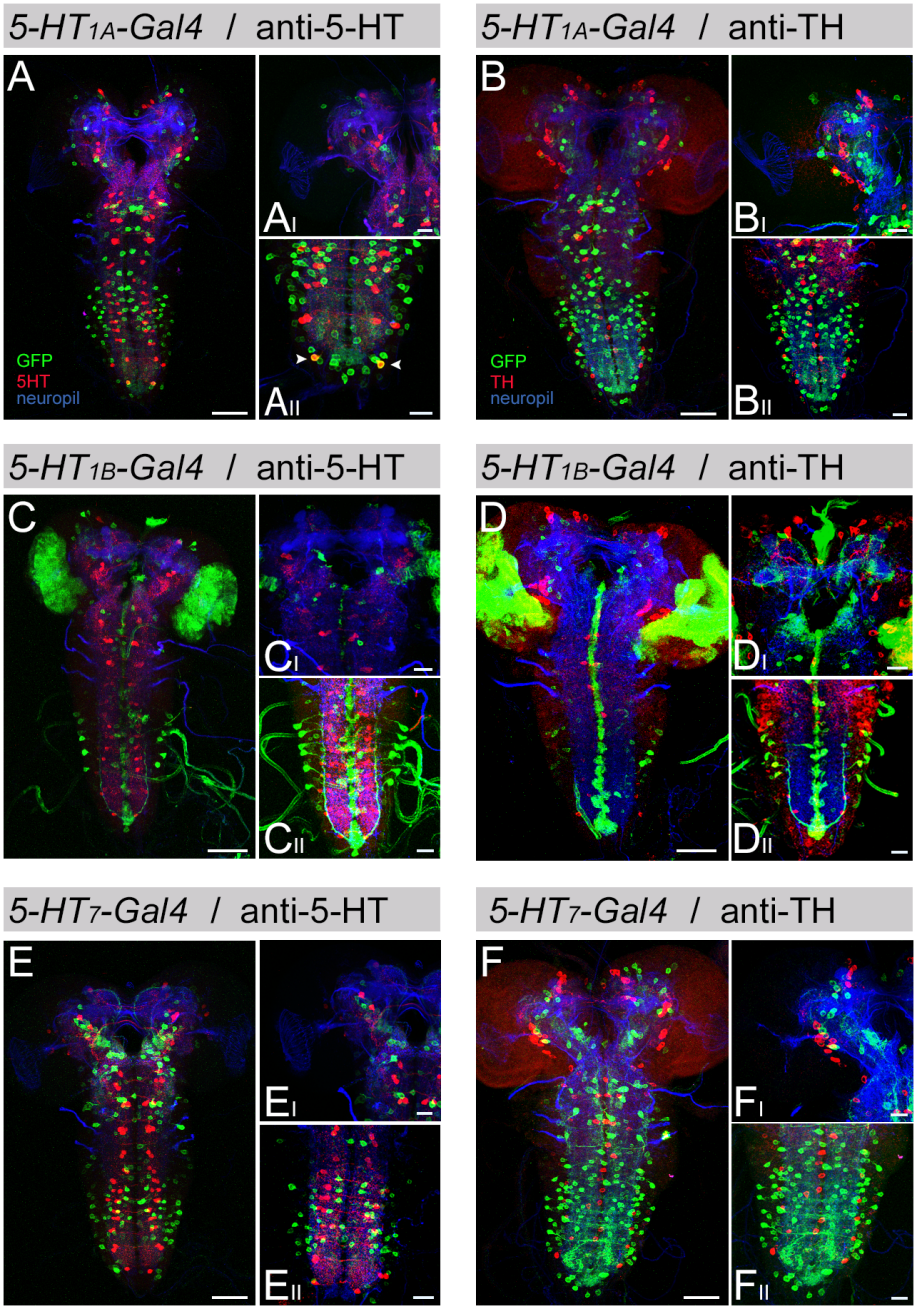
Co-expression of *5-HT*_*1A*_-, *5-HT*_*1B*_-, and *5-HT*_*7*_*-Gal4* with anti-5-HT and anti-TH. Expression patterns of *5-HT*_*1A*_-, *5-HT*_*1B*_-, and *5-HT*_*7*_*-Gal4* crossed with UAS-*mCD8*::*GFP* are visualized using a triple staining protocol. Anti-GFP (green) was used to label *Gal4* positive cells, anti-ChAT/anti-FasII (blue) was used as a reference labeling for the CNS, and anti-5-HT (red, in A, C, E) or anti-TH (red, in B, D, F) was used to identify 5-HT or dopaminergic cells, respectively. *5-HT*_*2A*_*-Gal4* was not included in this experiment due to the low number of cell bodies inside the CNS (Fig 1D). There was no co-staining detected between 5-HT receptor *Gal4* expression patterns and anti-TH (B, D, F). The same is true for the co-expression analysis with anti-5-HT (A, C, E); the only exception was a single pair of neurons in the terminal segment of the VNC labeled by *5-HT*_*1A*_*-Gal4* (arrowhead in A_II_). (A-F) show frontal views onto z-projections of the entire CNS (left) and representative z-projections of close-ups of one or both hemispheres and SOG (right top) and abdominal ganglion (right bottom). Scale bars: 50 μm (left) and 20 μm (right top and bottom).

In addition we used a second protocol developed at the HHMI Janelia research campus (https://www.janelia.org/project-team/flylight/protocols). Larval tissues were dissected, fixed and washed as described above. After blocking with 3% normal goat serum (Vector Laboratories, Burlingame, CA) in PBT for 1 hr, tissues were incubated for two days with first antibodies at 4°C. After multiple rinses in PBT, tissues were incubated 2 days at 4°C in the cocktail of secondary antibodies. Nervous systems were then washed two to three times in PBT, mounted on poly-L-lysine (Sigma-Aldrich) coated coverslips and then transferred to a coverslip staining jar (Electron Microscopy Sciences) to dehydrate through a graded ethanol series. Afterwards tissues were cleared in xylene, and mounted in DPX (Sigma). This protocol was used for data presented in Fig 1A_III_–1A_VII_, 1B_II_–1B_V_, 1B_VII_, 1C_I_–1C_III_, 1C_V_ and 1D_VII_.

Images were taken with Zeiss confocal laser microscopes LSM 550 and LSM 780. The resulting image stacks were projected and analyzed with ImageJ (NIH; https://imagej.nih.gov/ij/) software. Contrast and brightness adjustment as well as rotation and organization of images were performed in Photoshop (Adobe Systems Inc., San Jose, CA).

### Immunofluorescence antibodies

Anti-GFP (polyclonal/rabbit/A6455/Molecular Probes/1:1000), anti-GFP-FITC (polyclonal/goat/ ab6662/abcam/1:1000) and anti-DsRed (polyclonal/rabbit/632496/Clonetech/1:200) were used for visualizing *Gal4* lines expression patterns. Two different mouse antibodies for staining the neuropil (anti-ChAT (monoclonal/mouse/ChAT4B1/DSHB/1:100)) and the axonal tracts (anti-Fasciclin II (monoclonal/1d4 anti-Fas/DSHB/1:50)) were applied to provide landmarks within the larval CNS. 5-HT cells were visualized with anti-5-HT (polyclonal/rabbit/S5545/Sigma Aldrich /1:1000), dopaminergic cells with anti-TH (monoclonal/mouse/22941/Immuno-Star/1:500), respectively.

As secondary antibodies goat anti-rabbit IgG Alexa Fluor 488 (polyclonal/A11008/Molecular Probes/ 1:200), goat anti-mouse IgG Alexa Fluor 647 (polyclonal/A21235/Molecular Probes/1:200), goat anti-mouse IgG Alexa Fluor 405 (polyclonal/A31553/Molecular Probes/1:200), goat anti-mouse IgG Cy3 (polyclonal/A10521/Molecular Probes/1:200), and goat anti-rabbit IgG Cy5 (polyclonal/A10523/Molecular Probes/1:200) were used.

### Behavioral experiments

Five to six day old feeding third instar larvae were used for all behavioral experiments. The assays were performed either at 22°C or at 35°C using UAS-*hid*,*rpr* and UAS-*shi*^*ts*^ effector lines, respectively. In case of UAS-*shi*^*ts*^, larvae were additionally incubated for 2 min in a water bath at 37°C prior to behavioral experiments. For further details on experimental protocols we added the following description and refer to earlier studies [38–40, 43, 57, 59–62].

### Associative olfactory learning and memory

Experiments were conducted on test plates filled with a thin layer of 2.5% agarose containing either pure agarose or agarose plus reinforcer. We used 1.5 M sodium chloride (SALT) and 2.0 M D-fructose (FRU). As olfactory stimuli, we used 10 μl amyl acetate (AM, diluted 1:250 in paraffin oil) and benzaldehyde (BA, undiluted). Odorants were loaded into custom-made Teflon containers with perforated lids. Learning and memory were tested by exposing a first group of 30 animals to BA, while crawling on agarose medium containing sugar as a positive reinforcer or high salt concentration as a negative reinforcer. After 5 min, larvae were transferred to a fresh test plate in which they were allowed to crawl on pure agarose medium for 5 min while being exposed to AM. A second group of larvae received the reciprocal training. Immediately, after three training cycles, larvae were transferred onto test plates on which AM and BA were presented on opposite sides. Please note that for aversive olfactory learning and memory the test plate included the same high salt concentration as respective training plates. After 5 min, individuals were counted on the AM side (#AM), the BA side (#BA), and in a neutral zone. By subtracting the number of larvae on the BA side from the number of larvae on the AM side divided by the total number of counted individuals (#TOTAL), we calculated a preference index for each training group:

$${\text{PREF}}_{\text{AM}+/\text{BA}}=(\#\text{ AM - }\#\text{ BA})\;/\;\#\text{ TOTAL}$$ (1a)

$${\text{PREF}}_{\text{AM}/\text{BA}+}=(\#\text{ AM - }\#\text{ BA})\;/\;\#\text{ TOTAL}$$ (1b)

To measure specifically the effect of associative learning and memory we calculated the associative performance index (PI) as the difference in preference between the reciprocally trained larvae:

$$\text{PI}=({\text{PREF}}_{\text{AM}+/\text{BA}}\text{ - }{\text{PREF}}_{\text{AM}/\text{BA}+})\;/\;2$$ (2)

Negative PIs thus represent aversive olfactory learning and memory, whereas positive PIs indicate appetitive olfactory learning and memory. Division by 2 ensures scores are bound within (-1; 1).

### Chemosensory preference

For gustatory preference tests, 2.5% agarose (Sigma Aldrich Cat. No.: A5093, CAS No.: 9012-36-6) solution was boiled in a microwave oven and filled as a thin layer into test plates (85 mm diameter, Cat. No.: 82.1472, Sarstedt, Nümbrecht). After cooling, the agarose was removed from half of the plate. The empty half was filled with 2.5% agarose solution containing sodium chloride (SALT, Sigma Aldrich Cat. No.: S7653, CAS No.: 7647-14-5; 2.0 M and 1.5 M) and D-fructose (FRU, Sigma Aldrich Cat. No.: 47740, CAS No.: 57-48-7; 2.0 M). Assay plates were used at the same day shortly after preparation to avoid diffusion of the stimuli from one side to the other. Groups of 30 larvae were placed in the middle of the plate, allowed to crawl for 5 min, and then counted on the stimulus containing side, the agarose only side, and a neutral zone. The neutral zone covers 1 cm from top to bottom of the Petri dish between the left and right sides. It thereby separates both halfs and covers the transition from pure agarose to agarose plus gustatory stimulus. By subtracting the number of larvae on the pure agarose side (#nS) from the number of larvae on the stimulus side (#S) divided by the total number of counted larvae (#TOTAL), a preference index for the respective chemosensory stimulus was calculated:

PREF=(#S - #nS) /#TOTAL

Negative PREF values indicate avoidance, whereas positive PREF values represent attractiveness.

For olfactory preference tests, a similar assay was used except that olfactory stimuli were presented in custom-made Teflon containers with perforated lids presented on only pure agarose containing test plates. As olfactory stimuli amyl acetate (AM, Fluka Cat. No.: 46022; CAS No.: 628-63-7; diluted 1:250 in paraffin oil, Fluka Cat. No.: 76235, CAS No.: 8012-95-1) and benzaldehyde (BA, Fluka Cat. No.: 12010, CAS No.: 100-52-7; undiluted) were used.

### Acutely blocking synaptic output with *shibire*^*ts*^

To acutely block synaptic output of defined sets of cells we used UAS-*shi*^*ts1*^ [53]. Immediately before the experiment, larvae were incubated for 2 min in a water-bath at 37°C. The behavioral experiments were then performed as described before, at a restrictive temperature of about 35°C in a custom made chamber.

### Statistical methods

Statistical analysis and visualizations were done with R (version 2.15.2), R studio (version 0.98.1028) and Adobe Photoshop (version CC 2015.5). Behavioral data are visualized as box plots with medians (middle lines), 25% / 75% percentiles (box boundaries), and 10% / 90% percentiles (whiskers). Sample size in each case is n = 10–20. Kruskal-Wallis tests (KWT) were performed and, in case of significance, followed by Wilcoxon rank-sum tests (WRT); Bonferroni corrections were used for multiple comparisons as applicable (indicated with BWRT). Likewise, Wilcoxon signed-ranked tests (WST) were used to compare values against chance level. Visualization of statistical evaluations: if only n.s. is shown the initial KWT did not suggest for a difference between the three groups (p ≥ 0.05). When differences between each group are shown this provides the results of the BWRT as the initial KWT suggested for significance (p < 0.05). P values were rounded to three decimal places except for cases that would have resulted in zero. Here additional decimal places are given. Further details including raw data can be found in S1 Table.

## Results

### Expression patterns of four specific 5-HT receptor *Gal4* lines in the larval brain

To analyze the cellular anatomy of different 5-HT receptor *Gal4* lines in the CNS of third instar larvae of *Drosophila*, we crossed *5-HT*_*1A*_*-Gal4*, *5-HT*_*1B*_*-Gal4*, and *5-HT*_*7*_*-Gal4* with UAS-*mCD8*::*GFP*. We used an anti-GFP antibody to identify details of the cellular innervation and morphology and anti-Fasciclin II (FasII) / anti-Choline Acetyltransferase (ChAT) background staining (Fig 1), which label axonal tracts [63] and neuropils [64], respectively. The double labeling approach thus allows us to map the different 5-HT receptor *Gal4* expression patterns into a common neuropil reference. This approach was hitherto not applied to all of these lines at the larval stage. Please note, to clearly disentangle 5*HT*_*2A*_*-Gal4* dependent expression from marker expression we crossed *5-HT*_*2A*_*-Gal4* with UAS-*myr*::*tomato* and labelled specifically *Gal4* positive cells via an anti-DsRed antibody [65]. This is necessary as the enhancer trap piggyBac construct carries a “3xP3-EYFP” marker that is also recognized by the anti-GFP antibody [50, 66, 67]. The “3xP3-EYFP” marker is expressed under the control of the endogenous *Pax6* gene and thus on its own drives expression in the larval visual system and brain [68, 69].

#### 5-HT_1A_-Gal4

*5-HT*_*1A*_*-Gal4* expression was found throughout the larval CNS including both brain hemispheres and the ventral nerve cord (VNC) (Fig 1A). A closer inspection revealed innervation of the protocerebrum (arrow in Fig 1A_II_ and 1A_VI_), the AL (arrow in Fig 1A_IV_), to a lesser degree of the suboesophageal ganglion (SOG; Fig 1A_III_), and mushroom body calyx (CA; arrow in Fig 1A_V_). In contrast, the MB lobes and larval optic neuropil (LON) were not labeled (Fig 1A_VI_ and 1A_VII_). Characteristic for the *5-HT*_*1A*_*-Gal4* expression pattern was a row of cell bodies localized close to the midline in each thoracic and abdominal segment, (Fig 1A and 1A_I_) as well as a set of about 15 cell bodies dorsolateral of the CA innervating different protocerebral regions (Fig 1A_V_). In addition, several neurons were labled per thoracic and abdominal segment that leave the brain and potentially target the peripheral system of the larvae (arrows in Fig 1A). The obtained expression pattern is comparable to the ones described before [15, 29].

#### 5-HT_1B_-GAL4

*5-HT*_*1B*_*-Gal4* also showed expression in extended regions throughout the whole larval CNS, including both hemispheres and the VNC (Fig 1B). A closer inspection revealed innervation in cells at the tip and midline of the SOG (arrows in 1B_III_). A small number of only about 20, likely embryonic-born Kenyon cells (KC) [70], innervate the entire MB at the CA and at the surface of the MB peduncle, vertical and medial lobe (arrows in Fig 1B_V_ and 1B_VI_). No expression was found in the AL and LON (Fig 1B_IV_ and 1B_VII_). Besides the pronounced staining along the midline, a set of cells with somata in the lateral abdominal segments project to the terminal plexus of the VNC (arrows in Fig 1B_I_). These *5-HT*_*1B*_ positive cells were reported to express the peptide hormone leucokinin and play a role in larval turning behavior [71]. An additional characteristic of the *5-HT*_*1B*_*-Gal4* line is its massive expression laterally in both hemispheres that give rise to the adult optic lobes after metamorphosis (arrows in Fig 1B_II_ and 1B_VII_) [72]. Our results are in line with earlier findings of the expression patterns of *5-HT*_*1B*_ [19, 71].

#### 5-HT_7_-Gal4

*5-HT*_*7*_*-Gal4* expression was detected throughout the whole larval CNS including both hemispheres and the VNC (Fig 1C). We could not find innervation within the MB lobes and the LON by potential *5-HT*_*7*_ cells (Fig 1C_VI_ and 1C_VII_). Yet, innervation was detected in the VNC, SOG, AL and CA (arrows in Fig 1C_I_–1C_V_). Seven cells with somata in close proximity to the AL densely innervate the AL (Fig 1C_IV_). These are likely projection neurons as they follow the antennocerebral tract an innervate the CA (arrows in Fig 1C_V_). The peduncle of the MB was weakly innervated by a MB extrinsic neuron called BL neuron (right arrow in Fig 1C_VI_) [70]. In addition, *5-HT*_*7*_*-Gal4* shows dense innervation within the SOG potentially co-localizing with the 5-HT positive SE0 clusters [34, 48, 73]. These cells were reported to be involved in feeding and also in linking the external environment with the internal endocrine system [34, 73]. Overall, the observed expression is consistent with the one reported before [23].

#### 5-HT_2A_-Gal4

*5-HT*_*2A*_*-Gal4* showed an expression pattern that is remarkably different when compared to the other three driver lines (Fig 1D). There are nearly no somata detectable within the larval CNS (Fig 1D_I_–D_VII_). Yet, there is strong labelling within the SOG, likely by axonal terminals from neurons of the peripheral nervous system that enter the brain via different nerves (arrows in Fig 1D_III_). However, due to technical limitations we were not able to clearly localize these cells outside of the CNS. We are nonetheless convinced that these cells are *5-HT*_*2A*_*-Gal4* positive due to their initial description by Nichols [21] using the same *Gal4* line. In addition, we found a second type of brain innervation from the periphery, this time into the LON (arrow in Fig 1D_VII_). Please note that the larval MB was not innervated. Detectable staining was limited to adjacent fiber bundles (lower arrow in Fig 1D_VI_) and weak innervation of the dorsal protocerebrum (upper arrow in Fig 1D_VI_).

In summary, the four analyzed *Gal4* expression patterns suggest that 5-HT receptors may be expressed broadly throughout the brain in partially overlapping patterns including primary olfactory and gustatory brain centers (AL and SOG). Distinct receptors may be limited to particular types of cells like peripheral sensory neurons that project to the SOG (*5-HT*_*2A*_*-Gal4*) and MB Kenyon cells that are known to be involved in learning and memory (*5-HT*_*1B*_*-Gal4*). Yet, these results are based on *Gal4* expression patterns that may not represent the endogenous gene expression patterns and therefore have to be handled with care.

### Co-localization of 5-HT receptor *Gal4* lines with serotonin and Tyrosine-Hydroxylase

To examine if the larval 5-HT system is controlled in an auto-regulatory manner [74–76], we crossed *5-HT*_*1A*_-, *5-HT*_*1B*_-, and *5-HT*_*7*_*-Gal4* with UAS-*mCD8*::*GPF*, used the same primary antibody mixture as before (anti-ChAT/anti-FasII and anti-GPF), but added anti-5-HT (Fig 2A, 2C and 2E). We did not analyze *5-HT*_*2A*_*-Gal4* due to the low number of stained somata within the larval brain (Fig 1D), but rather afferent projections of peripheral neurons that have their somata outside the CNS. All three driver lines exhibited no co-labeling of *Gal4* positive and anti-5-HT staining (Fig 2A, 2C and 2E). This indicates that 5-HT receptors may localize mostly postsynaptically in the larva. This interpretation matches with earlier results that have analyzed 5-HT/5-HT receptor co-localization [23, 29]. Yet, we also found one exception: consistent with previous work [29] a single pair of *5-HT*_*1A*_ stained cells in the VNC co-localized with 5-HT immunolabeling (Fig 2A_II_, arrowheads).

In addition, we also analyzed if potential 5-HT receptor cells are dopaminergic. It was shown that different aminergic systems directly connect on each other. Octopaminergic neurons for example signal a specific aspect of sugar reinforcement directly onto dopaminergic neurons that express an α-adrenergic-like octopamine receptor called *OAMB* [77]. To address if the larval serotonergic system can directly act on dopaminergic neurons we applied a triple staining approach. We used anti-TH for the visualization of dopaminergic neurons, anti-GFP to label 5-HT receptor positive cells and anti-ChAT/anti-FasII for a reference staining of the larval CNS (Fig 2B, 2D and 2F). For the tested lines, *5-HT*_*1A*_-, *5-HT*_*1B*_-, and *5-HT*_*7*_*-Gal4*, we did not detect co-localization with dopamine synthesizing cells. Therefore, it is rather unlikely that the 5-HT system directly signals as a neurotransmitter on dopaminergic neurons.

### Ablation of *5-HT*_*2A*_*-Gal4* positive cells during development impairs aversive associative olfactory learning and memory

To examine if larvae lacking potential 5-HT receptor cells are able to associate an odor with a positive or negative gustatory stimulus, we utilized a well-established standard paradigm (reviewed in [38]). As olfactory stimuli we used benzaldehyde (BA) and amyl acetate (AM). As appetitive and aversive gustatory unconditioned stimuli we used fructose (FRU, 2.0 M) and high sodium chloride concentration (SALT, 1.5 M), respectively. This is possible, because ablation of potential 5-HT receptor cells did neither change naïve olfactory responses towards amyl acetate (diluted 1:250 in paraffin oil), benzaldehyde (undiluted), heptanol (undiluted), and nonanol (undiluted) (S1 Fig); nor gustatory responses to sodium chloride (2M and 1.5M for *5-HT*_*2A*_*-Gal4*), fructose (2M), arabinose (2M), and sorbitol (2M) (S2 and S3 Figs).

**Fig 3:**
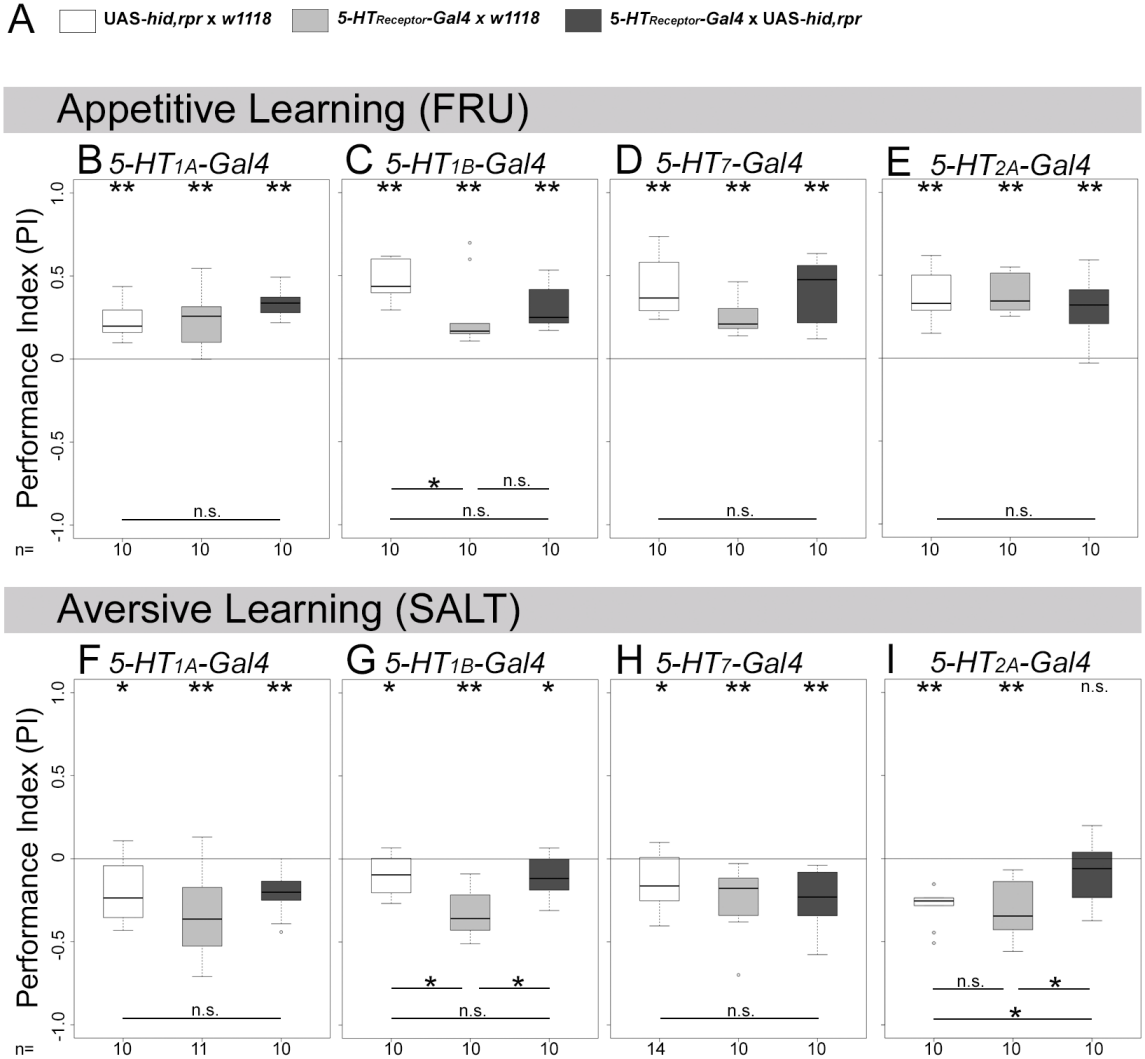
Ablation of potential *5-HT*_*2A*_ receptor cells throughout development impairs aversive olfactory learning and memory. *5-HT*_*1A*_-, *5-HT*_*1B*_-, *5-HT*_*7*_-, and *5-HT*_*2A*_*-Gal4* lines were crossed with UAS-*hid*,*rpr* to genetically induce apoptosis in potential 5-HT receptor cells. In addition, *Gal4* lines and UAS-*hid*,*rpr* were crossed with *w*^*1118*^ to obtain heterozygous genetic control larvae. (A) provides a color scheme for the three different groups used in each experiment. Appetitive olfactory learning and memory using fructose reinforcement is shown at the top (B-E). Aversive olfactory learning and memory is shown at the bottom (F-I). In most cases, experimental larvae and genetic control groups behaved similar. However, ablation of *5-HT*_*2A*_*-Gal4* positive cells specifically impaired aversive olfactory learning and memory (I), while leaving appetitive olfactory learning and memory intact (E). Sample size (n = 10–14) is indicated at the bottom of each box plot. Differences against zero are given at the top of each box plot. Differences between all three groups or individual groups are shown at the bottom of the panel. Visualization of statistical evaluations: if only n.s. is shown the initial Kruskal-Wallis test (KWT) did not suggest for a difference between the three groups (p > 0.05). When differences between each group are shown this provides the results of the Wilcoxon rank-sum tests with Bonferroni corrections (BWRT) as the initial KWT suggested for singnificance (p < 0.05). *** (p < 0.001), ** (p < 0.01), * (p < 0.05), n.s. (not significant p ≥ 0.05).

Ablation of *5-HT*_*1A*_*-Gal4*, *5-HT*_*1B*_*-Gal4*, *and 5-HT*_*7*_*-Gal4* positive cells throughout development did not change appetitive olfactory learning and memory (Fig 3B, KWT p = 0.061; Fig 3C, KWT p = 0.007; BWRT p = 0.189 compared to *5-HT*_*1B*_*-Gal4*/+ and p = 0.052 compared to UAS-*hid*,*rpr*/+; Fig 3D, KWT p = 0.061) as well as aversive olfactory learning and memory (Fig 3F, KWT p = 0.233; Fig 3G, KWT p = 0.006; BWRT p = 1.000 compared to *5-HT*_*1B*_*-Gal4*/+ and p = 0.016 compared to UAS-*hid*,*rpr*/+; Fig 3H, KWT p = 0.303).

Yet, *5-HT*_*2A*_*-Gal4*/UAS-*hid*,*rpr* experimental larvae showed impaired aversive olfactory learning and memory (Fig 3I; WST p = 0.131), which was significantly different compared to both genetic controls (Fig 3I; KWT p = 0.010; BWRT p = 0.021 compared to *5-HT*_*2A*_*-Gal4*/+, p = 0.034 compared to UAS-*hid*,*rpr*/+). Appetitive olfactory learning and memory did not differ among the three tested genotypes (Fig 3E; KWT p = 0.606). Repetition of the experiments with increased sample size gave rise to the same results (S4 Fig).

In summary, we thus conclude that *5-HT*_*1A*_-, *5-HT*_*1B*_-, and *5-HT*_*7*_*-Gal4* positive cells were not necessary for appetitive and aversive olfactory learning and memory. Yet, for *5-HT*_*2A*_*-Gal4* we got a different result; unlike appetitive olfactory learning and memory, aversive olfactory learning and memory was impaired.

### *5-HT*_*2A*_ receptor function throughout development is necessary for aversive olfactory learning and memory

To investigate if the memory impairment seen for *5-HT*_*2A*_*-Gal4*/UAS-*hid*,*rpr* experimental larvae was due to changes in the *5-HT*_*2A*_ receptor function, we next tested a homozygous hypomorphic *5-HT*_*2A*_ receptor mutant. In detail, Nichols [21] showed via quantitative real-time PCR analysis for this line that *5-HT*_*2A*_ expression is reduced by about 90% compared to wild type (*Oregon-R*) and *w*^*1118*^ flies.

**Fig 4:**
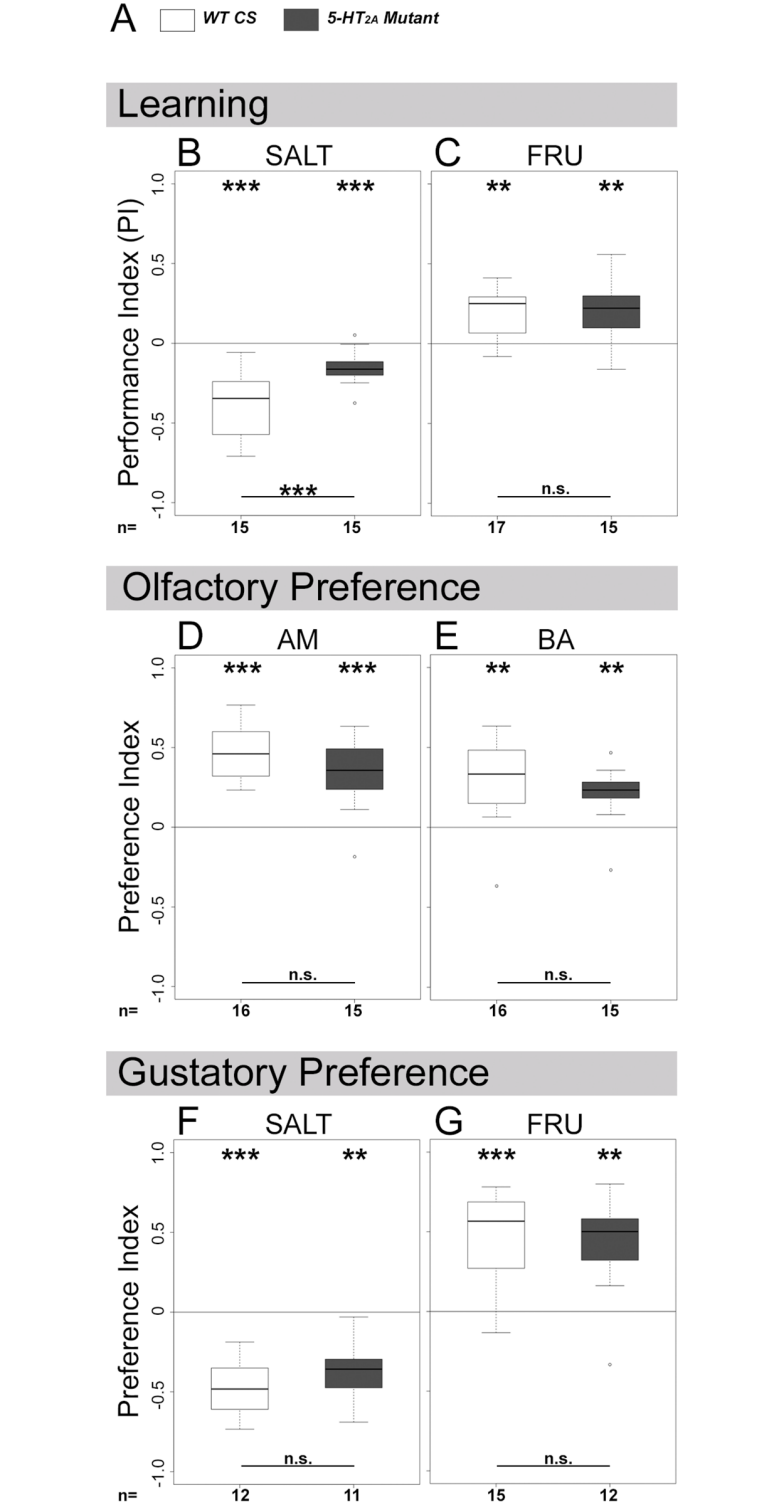
Impaired *5-HT*_*2A*_ receptor function throughout development impairs aversive olfactory learning and memory. Homozygous *5-HT*_*2A*_ receptor gene mutants and wild-type control larvae (*WT CS*) were used to analyze aversive (B) and appetitive (C) olfactory learning and memory, olfactory amyl acetate (AM, in D) and benzaldehyde (BA in E) preferences and gustatory sodium chloride (SALT, in F) and fructose (FRU, in G) preferences. (A) provides a color scheme for the two different groups used in each experiment. Whereas mutant larvae showed olfactory and gustatory preferences as well as appetitive olfactory learning and memory comparable to WT CS larvae, aversive olfactory learning and memory was significantly reduced. Sample size (n = 11–17) is indicated at the bottom of each box plot. Differences against zero are given at the top of each box plot. Differences between mutant and wild type larvae are shown at the bottom of the panel. *** (p < 0.001), ** (p < 0.01), * (p < 0.05), n.s. (not significant p ≥ 0.05).

Homozygous *5-HT*_*2A*_ mutant larvae showed a similar reduction for aversive olfactory learning and memory when compared to wild-type control (*WT CS*) larvae (Fig 4B; WRT p = 0.001). Again, the behavioral phenotype was specific for aversive olfactory learning and memory and did not affect appetitive olfactory learning and memory (Fig 4C; WRT p = 1.000 compared to *WT CS*). In addition, we performed control experiments with *5-HT*_*2A*_ mutant and *WT CS* larvae to test for proper olfactory and gustatory chemotaxis. This was necessary to exclude perturbing defects in task-relevant sensory-motor abilities. *5-HT*_*2A*_ receptor mutants displayed olfactory AM and BA preferences as well as gustatory SALT and FRU preferences that did not significantly differ from wild type controls (Fig 4D, 4E, 4F and 4G; WRT p = 0.173, p = 0.154, p = 0.148, p = 0.516, respectively).

Based on these results we conclude that *5-HT*_*2A*_ receptor function throughout development is necessary for aversive olfactory learning and memory using high salt concentration as a unconditioned stimulus.

### Ablation of the 5-HT/*5-HT*_*2A*_ receptor system throughout development specifically impairs aversive odor-salt learning and memory

**Fig 5:**
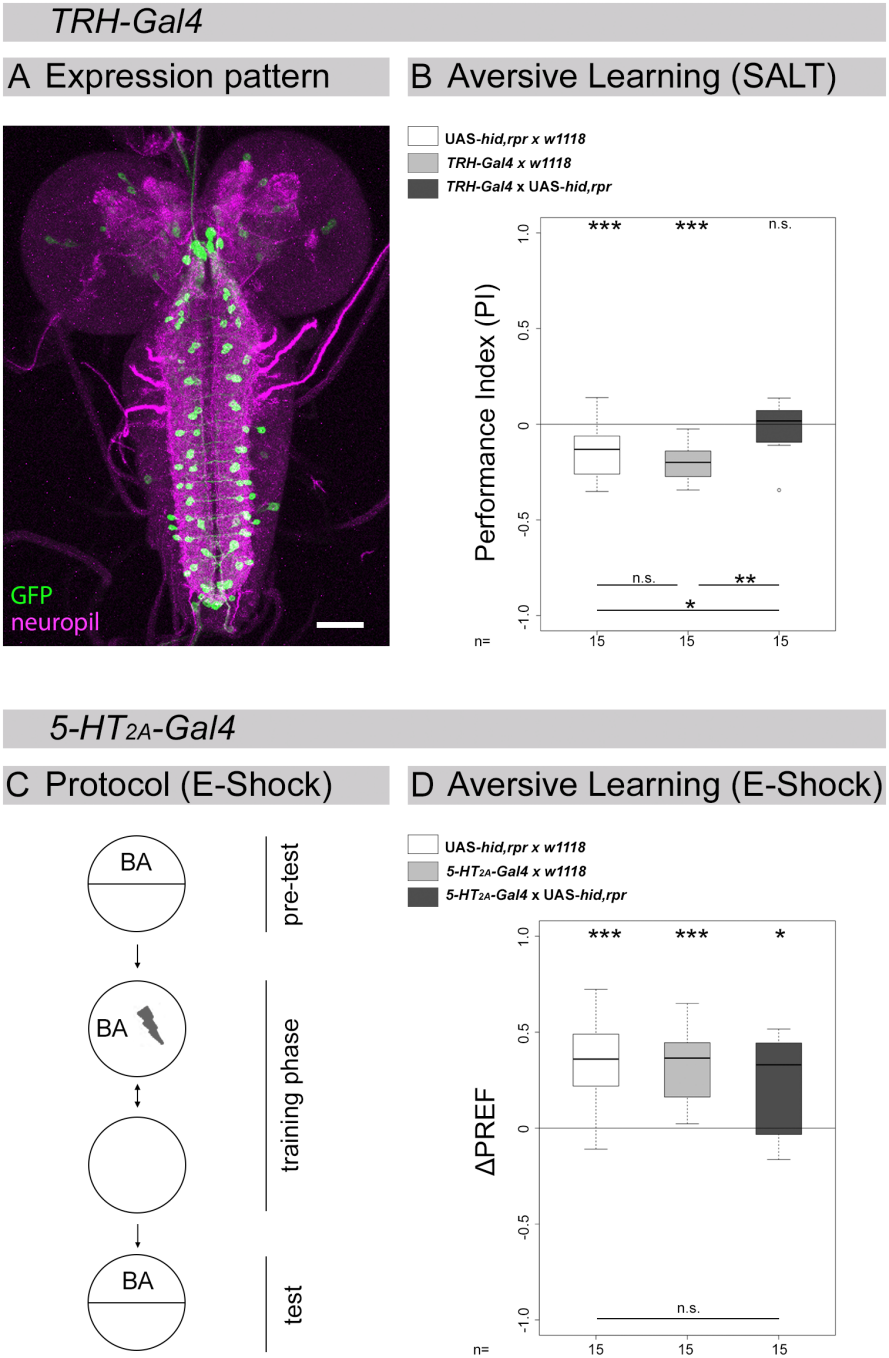
5-HT/*5-HT*_*2A*_ signaling throughout development specifically impairs odor-salt learning and memory. (A) The *TRH-Gal4* line was crossed with UAS-*mCD8*::*GFP* to visualize its expression pattern (green; anti-GFP staining) in addition to a reference labeling of the CNS (magenta; anti-ChAT/anti-FasII double-staining). (B) The *TRH-Gal4* line was crossed with UAS-*hid*,*rpr* to genetically induce apoptosis in 5-HT cells. In addition, the *Gal4* line and UAS-*hid*,*rpr* were crossed with *w*^*1118*^ to obtain heterozygous genetic control larvae. Above the panel a color scheme describes the three different groups used in the experiment. Ablation of 5-HT cells completely abolished aversive olfactory learning and memory reinforced by high salt concentration. (C) shows an overview on the experimental procedure that was used in larvae to test for odor-electric shock learning and memory. (D) Ablation of *5-HT*_*2A*_*-Gal4* positive cells via UAS-*hid*,*rpr* did not impair odor-electric shock learning and memory. Sample size (n = 15) is indicated at the bottom of each box plot. Differences against zero are given at the top of each box plot. Differences between the groups are shown at the bottom of the panel. Visualization of statistical evaluations: if only n.s. is shown the initial KWT did not suggest for a difference between the three groups (p > 0.05). When differences between each group are shown this provides the results of the BWRT as the initial KWT suggested for significance (p < 0.05). *** (p < 0.001), ** (p < 0.01), * (p < 0.05), n.s. (not significant p ≥ 0.05). Scale bar: 50 μm.

In an earlier study we had shown that larvae lacking 5-HT cells are able to establish an association between an odor and a punishing stimulus [48]. At first sight the behavioral phenotypes shown in Figs 3I and 4B, therefore, appear to be contradictory. Yet, in our initial experiments we used electric shock instead of high salt concentration as unconditioned stimulus. Thus, it is possible that manipulation of the 5-HT system does not affect aversive olfactory learning and memory in general but is rather restricted to gustatory high salt concentration. To investigate whether this is the case we utilized the same approach as used in Huser et al. (2012). We crossed the *TRH-Gal4* line [78] that expresses in most of the larval 5-HT cells (Fig 5A; [48]) as the *tryptophan hydroxylase (TRH)* gene was reported to catalyse the rate-limiting step of 5-HT synthesis from tryptophan to 5-hydroxy-tryptophan [1]. Experimental *TRH-Gal4*/UAS-*hid*,*rpr* larvae having most of their 5-HT cells ablated throughout development [48] did not show aversive odor-salt learning and memory (Fig 5B; WST p = 0.762). The behavior was different from both genetic controls (Fig 5B; KWT p = 0.002; BWRT p = 0.002 compared to *TRH-Gal4*/+, p = 0.038 compared to UAS-*hid*,*rpr*/+). The specificity for the unconditioned stimulus is further supported by a second experiment. Also *5-HT*_*2A*_*-Gal4*/UAS-*hid*,*rpr* experimental larvae that received odor-electric shock training performed similar as genetic controls and were not impaired in learning and memory (Fig 5C and 5D; KWT p = 0.470). We thus conclude that 5-HT/*5-HT*_*2A*_ receptor signaling throught development is necessary for aversive olfactory learning and memory reinforced by high salt concentration.

### Acute blockage of neuronal output of 5-HT and *5-HT*_*2A*_ receptor cells does not impair odor-salt learning and memory

To address if 5-HT function is acutely required for odor-salt learning and memory we used the temperature-sensitive dominant negative form of dynamin UAS-*shibire*^*ts*^ (*shi*^*ts*^) [53]. Thereby, we specifically disrupted synaptic vesicle recycling during training and testing but not during development.

**Fig 6:**
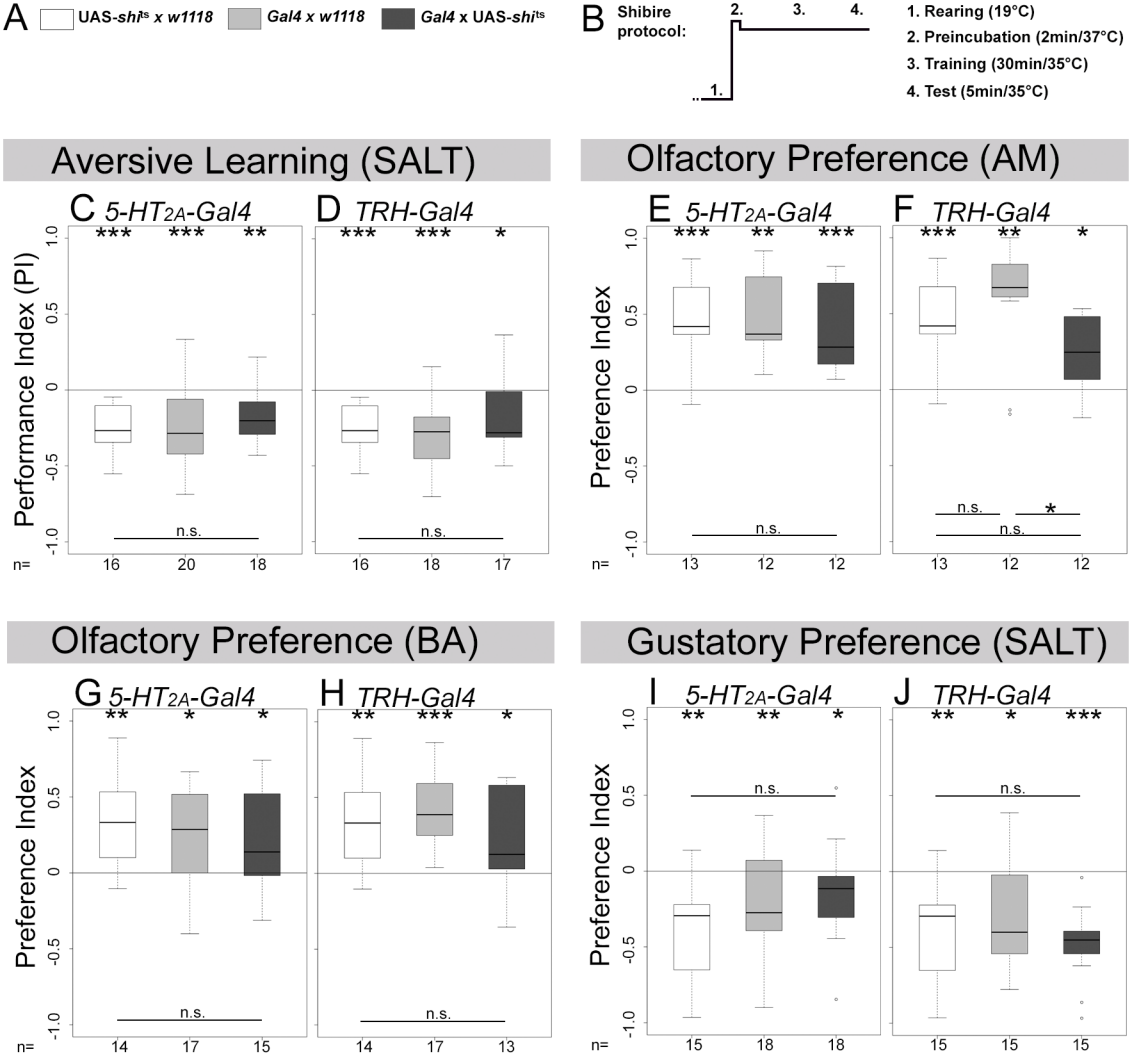
Acute blockage of synaptic output of 5-HT cells and potential *5-HT*_*2A*_ receptor cells does not affect chemosensory behavior. The *5-HT*_*2A*_*-Gal4* and *TRH-Gal4* lines were crossed with UAS-*shi*^*ts*^ to genetically interfere with synaptic transmission only during training and testing but not during development. In addition, the *Gal4* lines and UAS-*hid*,*rpr* were crossed with *w*^*1118*^ to obtain heterozygous genetic control larvae. (A) provides a color scheme for the different groups used in each experiment. (B) shows the temperature regime that was applied to block synaptic output at the restrictive temperature of 35°C specifically during training (30 min) and testing (5 min). Immediately before the experiment, larvae were incubated (2 min) in a water-bath at 37°C. Aversive olfactory learning and memory reinforced by high salt concentration (C and D), olfactory preferences for AM and BA (E–H) and gustatory preferences for SALT (I and J) were analyzed. In none of the cases experimental larvae behaved significantly different compared to both genetic control groups. We thus reason that blockage of synaptic output of 5-HT cells and *5-HT*_*2A*_*-Gal4* positive cells does not impair all tested chemosensory behaviors. Sample size (n = 12–18) is indicated at the bottom of each box plot. Differences against zero are given at the top of each box plot. Differences between all three groups or individual groups are shown at the bottom of the panel, except for SALT, where it is placed above the box plots. Visualization of statistical evaluations: if only n.s. is shown the initial KWT did not suggest for a difference between the three groups (p > 0.05). When differences between each group are shown this provides the results of the BWRT as the initial KWT suggested for singnificance (p < 0.05). *** (p < 0.001), ** (p < 0.01), * (p < 0.05), n.s. (not significant p ≥ 0.05).

An acute block of neurotransmission of *5-HT*_*2A*_*-Gal4* and *TRH-Gal4* positive cells did not affect odor-salt learning and memory (Fig 6C and 6D; WST p = 0.001 and p = 0.020). For both experiments we did not observe a difference between the three particular genotypes (Fig 6C and 6D; KWT p = 0.414 and p = 0.655).

In addition, experimental larvae were tested for olfactory AM and BA preferences and gustatory SALT preference. *5-HT*_*2A*_*-Gal4*/UAS-*shi*^*ts*^ were attracted by both odors (Fig 6E and 6G; WST p = 0.001 and p = 0.048) and avoided SALT (Fig 6I; WST p = 0.033). For all experiments there was no difference between experimental and control larvae (Fig 6E, 6G and 6I; KWT p = 0.560, KWT p = 0.660, KWT p = 0.127, respectively). The same results were observed with *TRH-Gal4* (Fig 6F, 6H and 6J; KWT p = 0.017; BWRT p = 0.029 compared to *TRH-Gal4*/+, p = 0.622 compared to UAS-*shi*^*ts*^/+; KWT p = 0.597, KWT p = 0.251, respectively). We thus conclude that acute blockage of neurotransmission of the 5-HT signaling system did not affect odor-salt learning and memory reinforced by high salt concentration as well as tested naïve chemosensory preferences.

## Discussion

### Ablation of 5-HT/*5-HT*_*2A*_ receptor signaling during development impairs aversive olfactory learning and memory reinforced by high salt concentration

Serotonin is a biogenic amine, an important neuroactive molecule within the CNS of several insect species. Serotonin, dopamine, histamine, octopamine, and tyramine are amines which have been extensively studied in *Drosophila* (reviewed in [1, 79, 80]. Each one of the five aminergic neuronal systems—including the serotonergic system—exhibits a stereotypic pattern of a small number of neurons that are widely distributed in the larval and adult CNS [1]. Aminergic neurons have attracted much attention in recent years. Thus, the detailed anatomy and various behavioral functions for many aminergic neurons were identified—in numerous cases even at single cell resolution (only focusing on the *Drosophila* larvae: [48, 57, 58, 62, 81]).

In contrast, much less is known on the receptor side. Due to the lack of specific antibodies and genetic tools to anatomically describe and functionally manipulate aminergic receptor cells only limited access and insight is given. Further, similar to vertebrates, for each amine different receptor genes were identified that in turn often couple to different signaling pathways and thereby provide a substrate for complex multi-dimensional functions (reviewed in [1, 82]). This complexity is additionally complicated by differences in the nomenclature (*5-HT*_*7*_ or *5-HT*_*7Dro*_ was also called *5-HT-dro1*; *5-HT*_*1A*_ or *5-HT*_*1ADro*_ was also called *5-HT-dro2A* [10]) and the identification of new receptor genes (*5-HT*_*2B*_ was only recently described [13]).

In this study we used state of the art *Gal4* lines to anatomically describe and functionally evaluate the role of *5-HT*_*1A*_, *5-HT*_*1B*,_ *5-HT*_*2A*_, and *5-HT*_*7*_ receptor cells with respect to larval chemosensory behaviors [21–23, 26, 27, 29–32, 71]. Due to the lack of specific antibodies this approach is limited as it is not possible to validate the correctness of each *Gal4* expression pattern. We found that putative 5-HT/*5-HT*_*2A*_ receptor signaling during development is necessary for odor-salt learning and memory. The finding is based on three mutually supportive results. First, ablation of potential *5-HT*_*2A*_ receptor cells throughout development specifically impairs odor-salt learning and memory (Fig 3). Second, *5-HT*_*2A*_ receptor mutant larvae show the same impairment specifically for odor-salt learning and memory (Fig 4). Third, ablation of 5-HT cells throughout development abolishes specifically odor-salt learning and memory (Fig 5), while leaving appetitive odor-sugar and aversive odor-electric-shock learning and memory intact [48]. Thereby, we describe for the first time a potential involvement of the 5-HT system in larval nervous system development underlying learning and memory. The mode of action, however, of the putative 5-HT/*5-HT*_*2A*_ receptor signaling is related to the development of the animal and independent of an acute neuronal function (Fig 6).

### 5-HT and *5-HT*_*2A*_ receptor cells are dispensable for most of the chemosensory behaviors tested

In addition, we found that potential *5-HT*_*1A*_, *5-HT*_*1B*_, and *5-HT*_*7*_ receptor cells are not necessary for any of the tested chemosensory behaviors (Fig 3, S1 and S2 Figs). However, given the missing verification for each of the three *Gal4* lines expression patterns this result has to be interpreted carefully. Moreover, *5-HT*_*2A*_ receptor cells were not required for olfactory and gustatory preferences and appetitive olfactory learning and memory (Fig 3, S1, S2, S3 and S4 Figs). Overall, it is remarkable that the obtained behavioral phenotypes are limited, particularly as several studies revealed that 5-HT receptors are essential for different aspects of larval behavior. For locomotion, for instance, *5-HT*_*1B*_ function was required within a small set of abdominal leucokinin positive neurons to suppress rearing [71]. RNAi-dependent knock-down of *5-HT*_*1B*_, *5-HT*_*2A*_, and *5-HT*_*7*_ receptors within the MB Kenyon cells increased the distance larvae crawled within 140 seconds [32]. Furthermore, *5-HT*_*2A*_ and *5-HT*_*7*_ receptor mutant larvae showed a reduced number of body-wall contractions [31]. Thus, it seems that larval locomotion requires the entire set of 5-HT receptors within the CNS to organize different aspects of the motor program. Nevertheless, 5-HT receptor function is not limited to locomotion. Pan-neuronal overexpression of *5-HT*_*1A*_, but not *5-HT*_*1B*_, *5-HT*_*2A*_, and *5-HT*_*7*_, increased the time larvae spend in the light [33]. In addition, *5-HT*_*2A*_ mutants and pan-neuronal knock-down of *5-HT*_*2A*_ reduces larval feeding [13]. This means that individual 5-HT receptors are also important for different kinds of larval behavior. For *5-HT*_*2A*_ this at least includes locomotion, feeding, and odor-salt learning and memory. Future work needs to address whether different sets of cells regulate distinct behavioral outputs or if a single set of cells has multiple functions. Interestingly, in our hands genetic interference with 5-HT receptor function did not affect olfactory and gustatory preferences nor appetitive olfactory learning and memory (Fig 3, S1, S2, S3 and S4 Figs). Thus, defects in certain aspects of locomotion did not prevent larvae from making chemosensory choices, at least within the test interval of 5 minutes that we have applied. Loss or knock-down of 5-HT receptor function does not completely compromise larval crawling and therefore still allows larvae to orientate within their chemosensory environment over longer time periods. In the future, recently established automated locomotion tracking techniques can be implemented that allow to reconstruct and evaluate larval runs and turns with high spatial and temporal resolution [83–87]. This will allow to evaluate if 5-HT receptor function is necessary for immediate chemosensory responses or if particular aspects of the chemosensory behavioral output are changed (for example turn rate and run distance).

Interestingly, although *5-HT*_*2A*_ function is required for feeding [13], larvae with missing *5-HT*_*2A*_ cells or reduced *5-HT*_*2A*_ expression are able to associate an odor with a food reward (Figs 3 and 4 and S4 Fig). This might suggest that at least two different systems process gustatory information in the larval CNS: one pathway modulates feeding and depends on 5-HT function, a second pathway is important for appetitive learning and memory and independent of 5-HT and its receptors. Indeed, it was shown that 5-HT function affects all feeding related motor patterns, including head tilting, mouth hook movement, and pharyngeal and esophagus movements [73, 88]. Whether the processing of the sweetness and/or the nutritional value of sugars contributes differently to these proposed pathways is not clear [45]. A similar dual system was recently also found for the processing of bitter quinine [89].

### The developmental effect of 5-HT/*5-HT*_*2A*_ receptor signaling is specific for odor-salt learning and memory

We have shown that 5-HT cells and *5-HT*_*2A*_ receptor function is necessary for odor-salt learning and memory during development. During learning olfactory stimuli are sensed by only 21 olfactory receptor neurons, which are housed in a single sensillum at the head of the larva, the dorsal organ [90–95]. The olfactory information from a given olfactory receptor neuron is further conveyed by 21 uniglomerular and 14 multiglomerular projection neurons from the AL to the lateral horn and the CA region of the MB [93, 96–99]. Here, intrinsic MB Kenyon cells provide a substrate for synaptic plasticity as olfactory information converges with gustatory reward and punishment signals from different sets of dopaminergic neurons [57, 62, 100, 101]. Further downstream only a limited number of about two dozen MB output neurons [70] transfer the information onto premotor centers to trigger learned behavior.

Given that 5-HT cells and *5-HT*_*2A*_ receptor function is dispensable for appetitive olfactory learning and memory (Figs 3 and 4 and S4 Fig) and given that 5-HT cells [48] as well as *5-HT*_*2A*_-*Gal4* positive cells (Fig 5) are not necessary for aversive odor-electric shock learning and memory, we assume that 5-HT/*5-HT*_*2A*_ receptor signaling is specifically necessary for salt reinforcement processing. Otherwise, due to the overlapping neuronal circuits (including the olfactory circuit, the mushroom body, and the premotor and motor centers), more general impairments in learning and memory would have occurred.

Where in the reinforcing pathway does 5-HT/*5-HT*_*2A*_ receptor signaling become effective? Unfortunately, very little is known about the neuronal pathways signaling aversive salt reinforcement. In *Drosophila* larvae there is no evidence for direct 5-HT input onto the MB [48]. In adults, however, 5-HT positive neurons innervate the MB lobes and CA, termed DPM (dorsal paired medial) and CSD (contralaterally projecting serotonin-immunoreactive deutocerebral) neuron [102, 103]. Yet, the DPM neuron is not present at the larval stage [70] and the CSD neuron only innervates both AL and the lateral protocerebrum but misses the adult specific innervation of the CA and lateral horn [103].

Instead, *5-HT*_*2A*_*-Gal4* line expression nearly exclusively innervates the SOG (Fig 1). Most of the innervation comes from cells having their somata outside the CNS. Unfortunately, due to technical limitations we were not able to clearly localize them. However, *TRH-Gal4* has a pronounced expression within the SOG (Fig 5 [48]). Thus, although we favor the hypothesis that 5-HT/*5-HT*_*2A*_ receptor signaling, which is necessary for salt reinforcement can be attributed to the SOG, future work is needed for validation. In addition, we cannot exclude that the *5-HT*_*2A*_ receptor *Gal4* expression pattern may be misleading, given that we were able to show that acute blockage of 5-HT/*5-HT*_*2A*_ receptor neurotransmission does not impair odor-salt learning and memory (Fig 6). Thus, neuromodulatory or developmental processes may underlie 5-HT/*5-HT*_*2A*_ receptor function. Indeed, *5-HT*_*2A*_ receptor gene expression pattern markedly changes over larval development [21]. Therefore, it is possible that the impairment of odor-salt learning and memory is based on cellular functions, which are no longer included in the expression pattern of third instar larvae that we used for our analysis. This renders a localization of the cellular effects rather difficult.

### 5-HT and *5-HT*_*2A*_ receptor signaling regulate developmental processes

Before adopting their roles as neurotransmitters in the mature CNS, neuroactive substances function in the establishment of neural networks [104]. This is also true for 5-HT that can serve as a neurotransmitter and a neuromodulator in all animal phyla studied (reviewed in [105]). In vertebrates it was shown that 5-HT modulates different developmental events, including neuronal migration, cell differentiation, and synaptogenesis (reviewed in [106]). In invertebrates 5-HT regulates—among other processes—cell division in mollusca and the development of the AL during metamorphosis in moths [107, 108]. For *Drosophila* larvae it was shown that dopa decarboxylase mutants that are devoid of 5-HT and dopamine increase the extent of branching of 5-HT projections to the proventriculus and midgut, similar to larvae in which neuronal 5-HT synthesis was constitutively knocked-down [76, 109]. In addition, constitutively knocked-down 5-HT synthesis showed an increased number and size of varicosities in 5-HT fiber projections to the proventriculus [76]. This kind of autoregulation for the organization of 5-HT varicosities was also described in the larval CNS in 5-HT neurons of the A7 segment of the abdominal ganglion [76, 110].

Developmental functions of 5-HT signaling are not limited to larval stages but also include embryonic development. High levels of *5-HT*_*2A*_ receptor expression occur already at 3 hours of embryonic development and match with the seven-stripe pattern of the pair-rule gene *Fushi tarazu* [16]. 5-HT signaling through the *5-HT*_*2A*_ receptor triggers changes in cell adhesiveness that are necessary for normal germband extension during gastrulation [111, 112].

In summary, there is good evidence that 5-HT/*5-HT*_*2A*_ receptor signaling serves several developmental functions, including the regulation of neuronal connectivity in addition to their classic role in synaptic transmission. Future work needs to address how this might affect unconditioned stimulus processing using high salt concentration. In the longer run this may uncover 5-HT dependent organizational principles of reinforcement processing shared with adult *Drosophila* and other insects.

## Supporting information

S1 Fig Ablation of potential 5-HT receptor cells does not alter olfactory preferences towards four different odors. *5-HT*_*1A*_-, *5-HT*_*1B*_-, *5-HT*_*7*_-, and *5-HT*_*2A*_*-Gal4* lines were crossed with UAS-*hid*,*rpr* to genetically induce apoptosis in potential 5-HT receptor cells. In addition, *Gal4* lines and UAS-*hid*,*rpr* were crossed with *w*^*1118*^ to obtain heterozygous genetic control larvae. (A) provides a color scheme for the three different groups used in each experiment. Naïve olfactory preferences for amyl acetate (AM, in B, C, D, E), benzaldehyde (BA, in F, G, H, I), heptanol (HEP, in J, K, L, M), and nonanol (NON, in N, O, P, Q) were analyzed. In none of the cases experimental larvae behaved significantly different from both genetic control groups. We thus reason that ablation of potential 5-HT receptor cells does not impair the ability of the larvae to detect olfactory cues. The sample size (n = 11–16) is indicated under each box plot. Differences against random distribution are given at the top of each box plot. Differences between all three groups or individual groups are shown at the bottom of the panel. *** (p < 0.001), ** (p < 0.01), * (p < 0.05), n.s. (not significant p ≥ 0.05).
(TIF)

S2 Fig Ablation of potential 5-HT receptor cells does not alter gustatory preferences towards four different tastants. *5-HT*_*1A*_-, *5-HT*_*1B*_-, *5-HT*_*7*_-, and *5-HT*_*2A*_*-Gal4* lines were crossed with UAS-*hid*,*rpr* to genetically induce apoptosis in potential 5-HT receptor cells. In addition, *Gal4* lines and UAS-*hid*,*rpr* were crossed with *w*^*1118*^ to obtain heterozygous genetic control larvae. (A) provides a color scheme for the three different groups used in each experiment. Naïve gustatory preferences for sodium chloride (SALT, in B, C, D, E), fructose (FRU, in F, G, H, I), arabinose (ARA, in J, K, L, M), and sorbitol (SOR, in N, O, P, Q) were analyzed. In none of the cases experimental larvae behaved significantly different to both genetic control groups. We thus reason that ablation of potential 5-HT receptor cells does not impair the ability of the larvae to detect gustatory stimuli. Sample size (n = 13–20) is indicated under each box plot. Differences against random distribution are given at the top of each panel. Differences between all three groups or individual groups are shown at the bottom of the panel, except for SALT, where it is placed above the box plots. *** (p < 0.001), ** (p < 0.01), * (p < 0.05), n.s. (not significant p ≥ 0.05).
(TIF)

S3 Fig Ablation of potential 5-HT_2A_ receptor cells does not alter gustatory preferences towards 1.5 M sodium chloride. *5-HT*_*2A*_*-Gal4* was crossed with UAS-*hid*,*rpr* to genetically induce apoptosis in potential 5-HT_2A_ receptor cells. In addition, the *Gal4* line and UAS-*hid*,*rpr* were crossed with *w*^*1118*^ to obtain heterozygous genetic control larvae. Naïve gustatory preferences for 1.5 M sodium chloride (SALT) was analyzed. Experimental larvae behaved at the same level as both genetic control groups. We thus reason that ablation of potential 5-HT_2A_ receptor cells does not impair the ability of the larvae to detect 1.5 M sodium chloride. Sample size (n = 15) is indicated under each box plot. Differences against random distribution are given at the top of each panel. Differences between all three groups or individual groups are shown above the box plots. n.s. indicates that the initial KWT did not suggest for a difference between the three groups (p > 0.05). *** (p < 0.001), ** (p < 0.01), n.s. (not significant p ≥ 0.05).
(TIF)

S4 Fig Ablation of potential *5-HT*_*2A*_ receptor cells throughout development impairs aversive olfactory learning and memory. *5-HT*_*2A*_*-Gal4* was crossed with UAS-*hid*,*rpr* to genetically induce apoptosis in potential 5-HT_2A_ receptor cells. In addition, the *Gal4* line and UAS-*hid*,*rpr* were crossed with *w*^*1118*^ to obtain heterozygous genetic control larvae. (A) provides a color scheme for the three different groups used in each experiment. (B) Appetitive olfactory learning and memory using fructose reinforcement is shown at the top. (C) Aversive olfactory learning and memory is shown at the bottom. (B) For appetitive olfactory learning experimental larvae and genetic control groups behaved similar. Yet, ablation of *5-HT*_*2A*_*-Gal4* positive cells throughout development specifically impaired aversive olfactory learning and memory (C). Sample size (n = 17–20) is indicated at the bottom of each box plot. Differences against zero are given at the top of each box plot. Differences between all three groups or individual groups are shown at the bottom of the panel. Visualization of statistical evaluations: if only n.s. is shown the initial KWT did not suggest for a difference between the three groups (p ≥ 0.05). When differences between each group are shown this provides the results of the BWRT as the initial KWT suggested for significance (p > 0.05). *** (p < 0.001), ** (p < 0.01), * (p < 0.05), n.s. (not significant p ≥ 0.05).
(TIF)

S1 Table Raw data behavior. (XLSX)

S1 File Supplemental material and methods. (PDF)

S2 File Movie 1: *5-HT*_*1A*_*-GAL4* expression. (AVI)

S3 File Movie 2: *5-HT*_*1A*_*-GAL4* expression (GFP). (AVI)

S4 File Movie 3: *5-HT*_*1B*_*-GAL4* expression. (AVI)

S5 File Movie 4: *5-HT*_*1B*_*-GAL4* expression (GFP). (AVI)

S6 File Movie 5: *5-HT*_*7*_*-GAL4* expression. (AVI)

S7 File Movie 6: *5-HT*_*7*_*-GAL4* expression (GFP). (AVI)

S8 File Movie 7: *5-HT*_*2A*_*-GAL4* expression. (AVI)

S9 File Movie 8: *5-HT*_*2A*_*-GAL4* expression (dsRed). (AVI)
