# Supplementary figures and images for: Anatomy and behavioral function of serotonin receptors in Drosophila melanogaster larvae
Source: PLoS One. 2017 Aug 4;12(8):e0181865. doi: 10.1371/journal.pone.0181865 (PMC5544185; doi:10.1371/journal.pone.0181865)

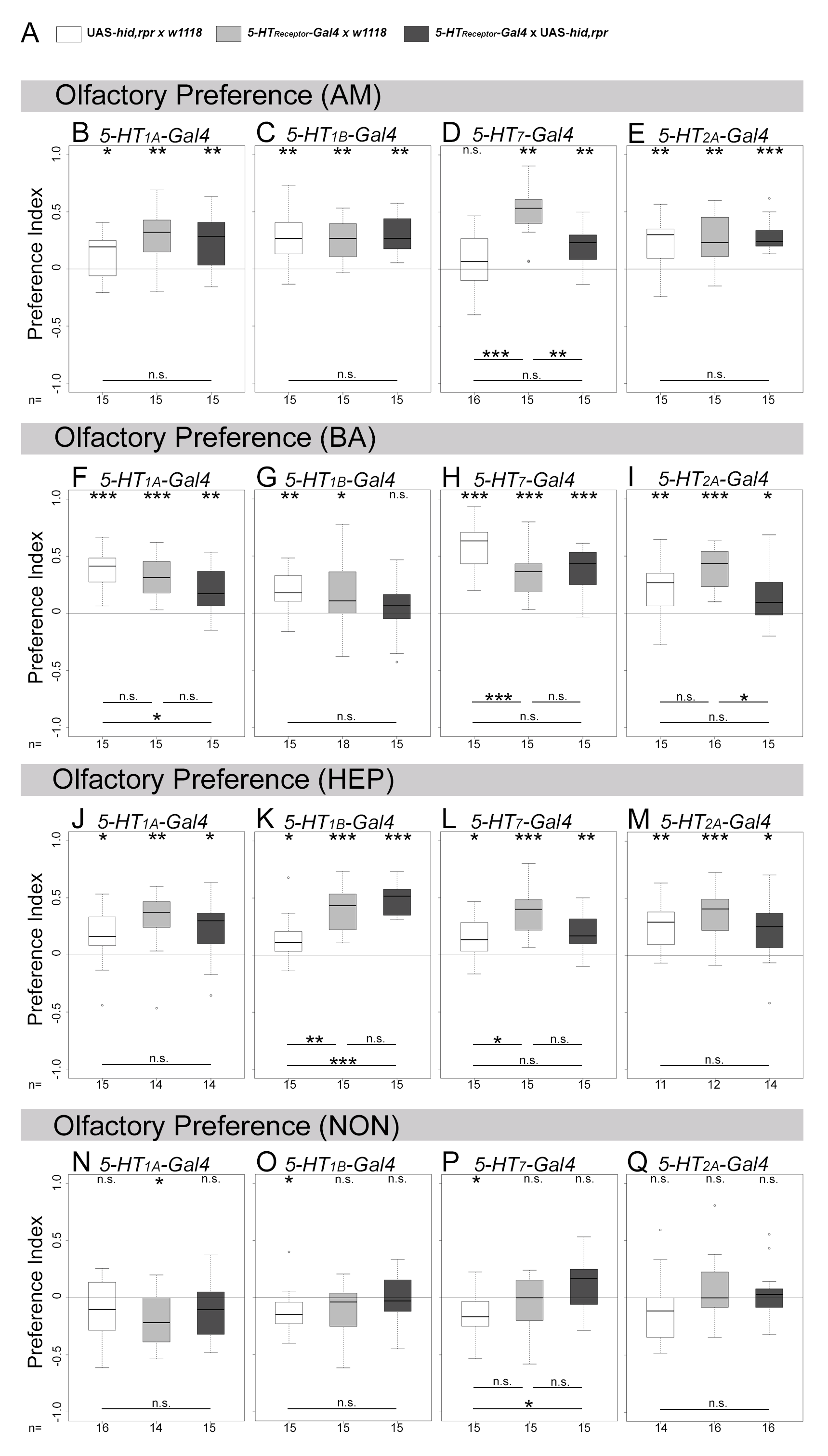

Supplement: S1 Fig — 5-HT1A-, 5-HT1B-, 5-HT7-, and 5-HT2A-Gal4 lines were crossed with UAS-hid,rpr to genetically induce apoptosis in potential 5-HT receptor cells. In addition, Gal4 lines and UAS-hid,rpr were crossed with w1118 to obtain heterozygous genetic control larvae. (A) provides a color scheme for the three different groups used in each experiment. Naïve olfactory preferences for amyl acetate (AM, in B, C, D, E), benzaldehyde (BA, in F, G, H, I), heptanol (HEP, in J, K, L, M), and nonanol (NON, in N, O, P, Q) were analyzed. In none of the cases experimental larvae behaved significantly different from both genetic control groups. We thus reason that ablation of potential 5-HT receptor cells does not impair the ability of the larvae to detect olfactory cues. The sample size (n = 11–16) is indicated under each box plot. Differences against random distribution are given at the top of each box plot. Differences between all three groups or individual groups are shown at the bottom of the panel. *** (p < 0.001), ** (p < 0.01), * (p < 0.05), n.s. (not significant p ≥ 0.05). (TIF) [file pone.0181865.s001.tif]

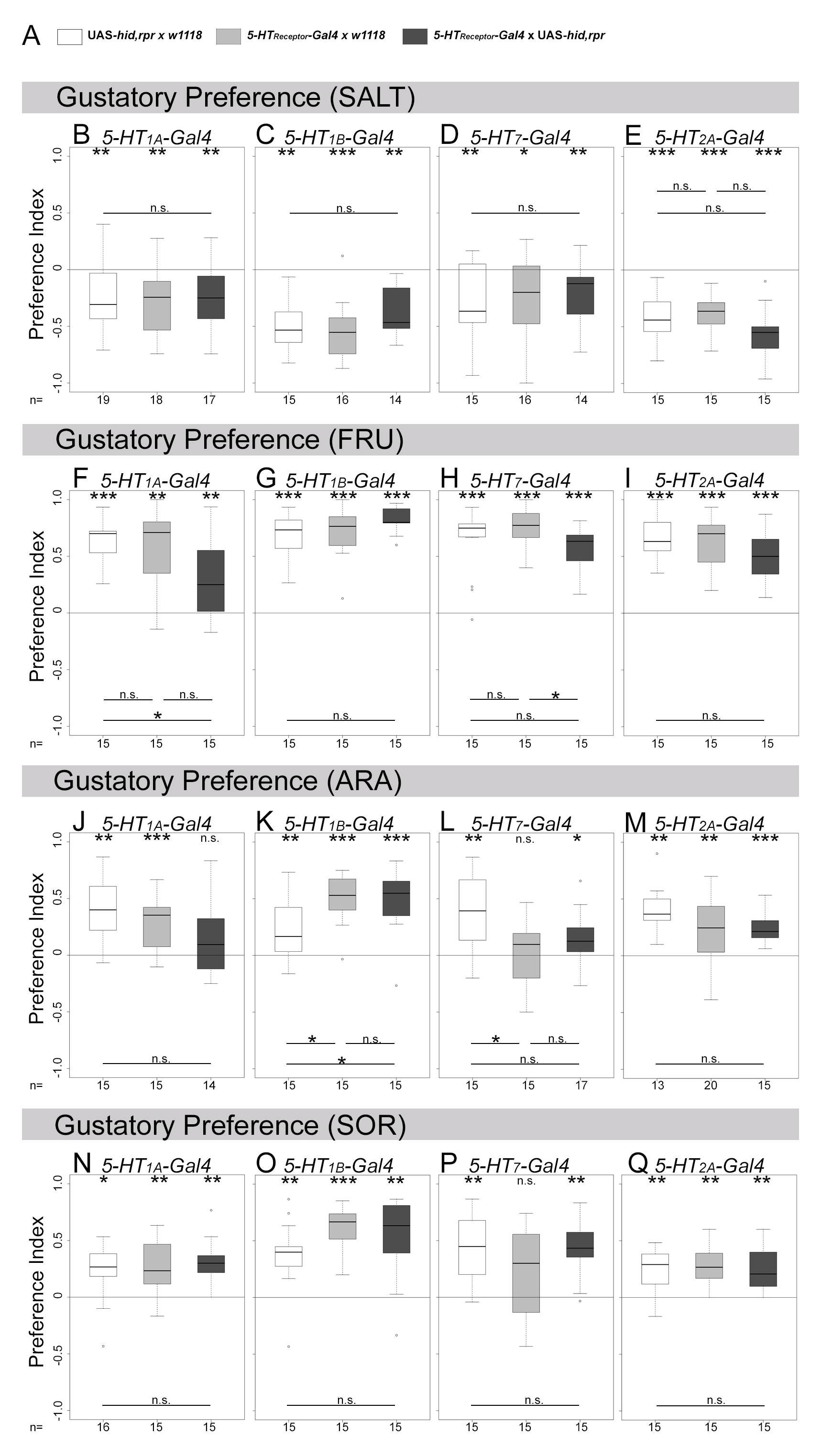

Supplement: S2 Fig — 5-HT1A-, 5-HT1B-, 5-HT7-, and 5-HT2A-Gal4 lines were crossed with UAS-hid,rpr to genetically induce apoptosis in potential 5-HT receptor cells. In addition, Gal4 lines and UAS-hid,rpr were crossed with w1118 to obtain heterozygous genetic control larvae. (A) provides a color scheme for the three different groups used in each experiment. Naïve gustatory preferences for sodium chloride (SALT, in B, C, D, E), fructose (FRU, in F, G, H, I), arabinose (ARA, in J, K, L, M), and sorbitol (SOR, in N, O, P, Q) were analyzed. In none of the cases experimental larvae behaved significantly different to both genetic control groups. We thus reason that ablation of potential 5-HT receptor cells does not impair the ability of the larvae to detect gustatory stimuli. Sample size (n = 13–20) is indicated under each box plot. Differences against random distribution are given at the top of each panel. Differences between all three groups or individual groups are shown at the bottom of the panel, except for SALT, where it is placed above the box plots. *** (p < 0.001), ** (p < 0.01), * (p < 0.05), n.s. (not significant p ≥ 0.05). (TIF) [file pone.0181865.s002.tif]

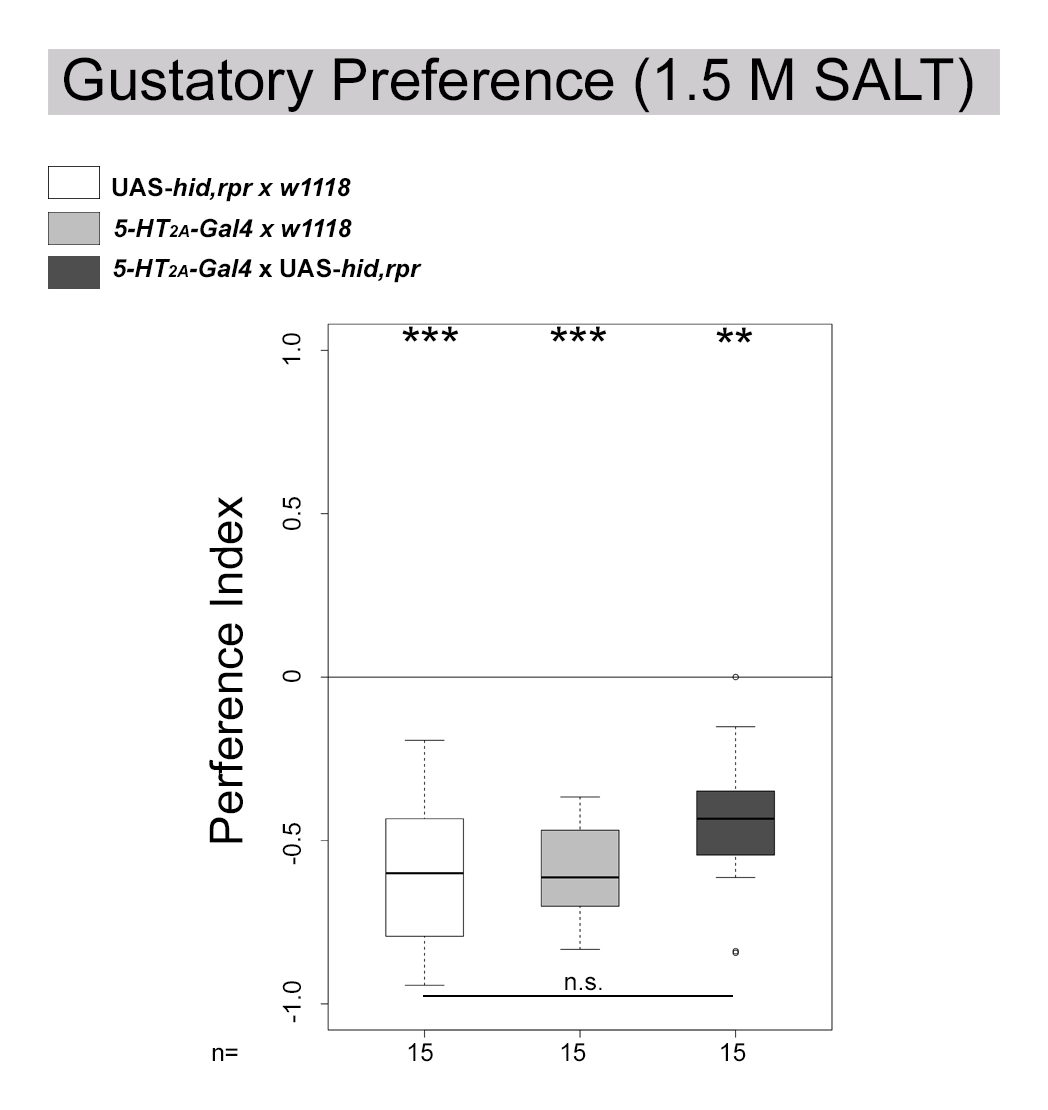

Supplement: S3 Fig — 5-HT2A-Gal4 was crossed with UAS-hid,rpr to genetically induce apoptosis in potential 5-HT2A receptor cells. In addition, the Gal4 line and UAS-hid,rpr were crossed with w1118 to obtain heterozygous genetic control larvae. Naïve gustatory preferences for 1.5 M sodium chloride (SALT) was analyzed. Experimental larvae behaved at the same level as both genetic control groups. We thus reason that ablation of potential 5-HT2A receptor cells does not impair the ability of the larvae to detect 1.5 M sodium chloride. Sample size (n = 15) is indicated under each box plot. Differences against random distribution are given at the top of each panel. Differences between all three groups or individual groups are shown above the box plots. n.s. indicates that the initial KWT did not suggest for a difference between the three groups (p > 0.05). *** (p < 0.001), ** (p < 0.01), n.s. (not significant p ≥ 0.05). (TIF) [file pone.0181865.s003.tif]

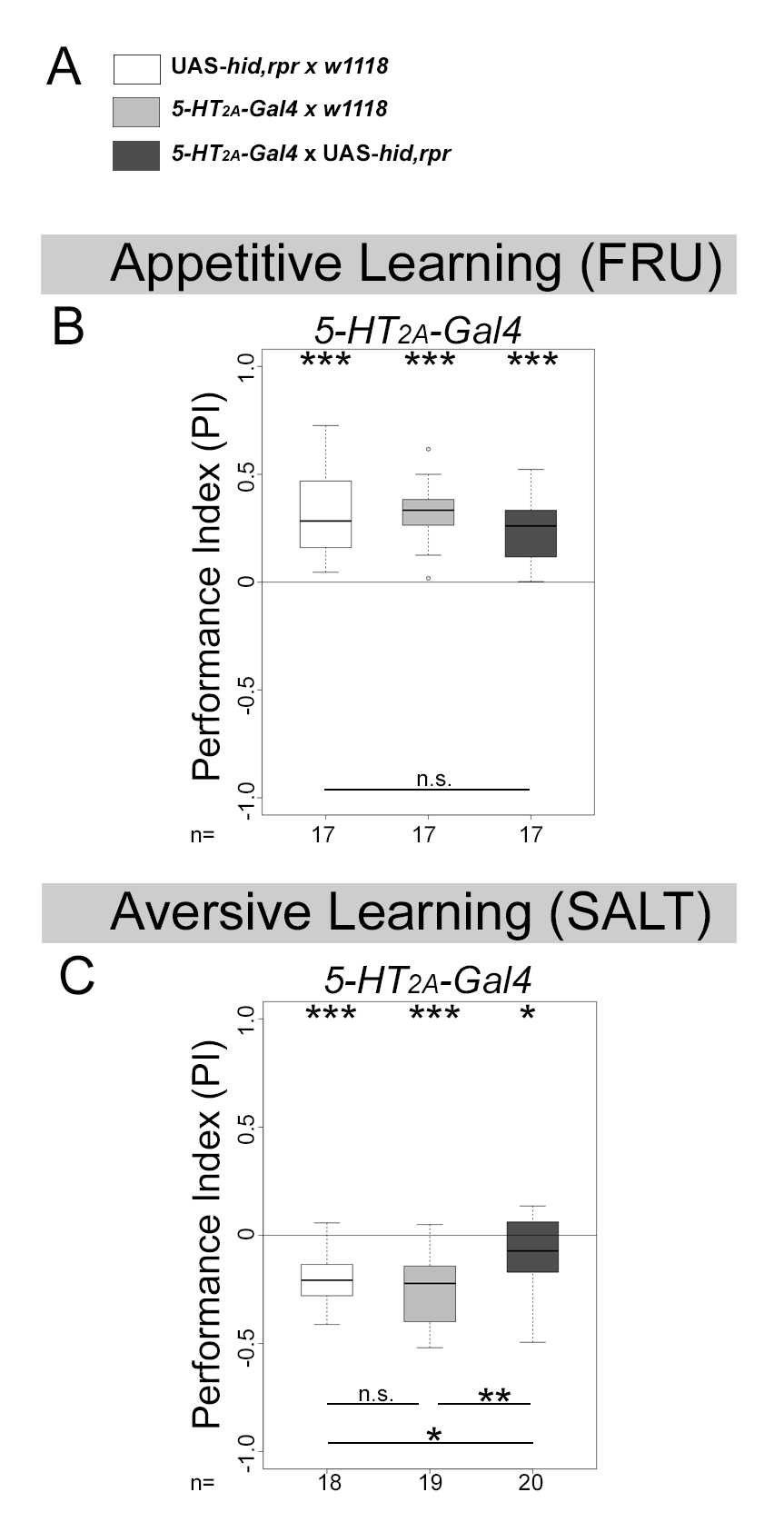

Supplement: S4 Fig — 5-HT2A-Gal4 was crossed with UAS-hid,rpr to genetically induce apoptosis in potential 5-HT2A receptor cells. In addition, the Gal4 line and UAS-hid,rpr were crossed with w1118 to obtain heterozygous genetic control larvae. (A) provides a color scheme for the three different groups used in each experiment. (B) Appetitive olfactory learning and memory using fructose reinforcement is shown at the top. (C) Aversive olfactory learning and memory is shown at the bottom. (B) For appetitive olfactory learning experimental larvae and genetic control groups behaved similar. Yet, ablation of 5-HT2A-Gal4 positive cells throughout development specifically impaired aversive olfactory learning and memory (C). Sample size (n = 17–20) is indicated at the bottom of each box plot. Differences against zero are given at the top of each box plot. Differences between all three groups or individual groups are shown at the bottom of the panel. Visualization of statistical evaluations: if only n.s. is shown the initial KWT did not suggest for a difference between the three groups (p ≥ 0.05). When differences between each group are shown this provides the results of the BWRT as the initial KWT suggested for significance (p > 0.05). *** (p < 0.001), ** (p < 0.01), * (p < 0.05), n.s. (not significant p ≥ 0.05). (TIF) [file pone.0181865.s004.tif]
