# Supplementary material for: Anatomy and behavioral function of serotonin receptors in Drosophila melanogaster larvae
Source: PLoS One. 2017 Aug 4;12(8):e0181865. doi: 10.1371/journal.pone.0181865 (PMC5544185; doi:10.1371/journal.pone.0181865)
Supplement: S1 File — (PDF) [file pone.0181865.s006.pdf]

Supporting information

Materials and Methods

Flies

Flies were maintained on standard *Drosophila* medium at 25°C or 19°C under 12h light /dark conditions. All 5-HT receptor specific lines, *5-HT$_{1A}$-Gal4* [29], *5-HT$_{1B}$-Gal4* [49] (Bloomington Stock Center no. 24240), *5-HT$_{2A}$-Gal4* [21, 50] (Bloomington Stock Center no. 19367), *5-HT$_{7}$-Gal4* [23], were kindly provided by Charles Nichols. Note that the *5-HT$_{2A}$-Gal4* construct is an enhancer trap piggyBac construct in the *5-HT$_{2A}$* locus that reduces its expression by nearly 90% [21]. For behavioral experiments, the effector line UAS-*hid,rpr* [51, 52] was used. w^{1118} flies (kindly provided by Martin Heisenberg) were crossed with UAS- and *Gal4* lines to obtain heterozygous controls. In all cases third instar larvae were analyzed.

Chemosensory preference

For gustatory preference tests, 2.5% agarose (Sigma Aldrich Cat. No.: A5093, CAS No.: 9012-36-6) solution was boiled in a microwave oven and filled as a thin layer into test plates (85 mm diameter, Cat. No.: 82.1472, Sarstedt, Nümbrecht). After cooling, the agarose was removed from half of the plate. The empty half was filled with 2.5% agarose solution containing sodium chloride (SALT, Sigma Aldrich Cat. No.: S7653, CAS No.: 7647-14-5; 2.0 M and 1.5 M), D-fructose (FRU, Sigma Aldrich Cat. No.: 47740, CAS No.: 57-48-7; 2.0 M), D-sorbitol (SORB, Sigma Aldrich Cat. No.: W302902, CAS No.: 50-70-4; 2.0 M), or D-arabinose (ARA, Sigma Aldrich Cat. No.: 10850, CAS No.: 10323-20-3; 2.0 M). Assay plates were used at the same day shortly after preparation to avoid diffusion of the stimuli from one side to the other. Groups of 30 larvae were placed in the middle of the plate, allowed to crawl for 5 min, and then counted on the stimulus containing side, the agarose only side, and a neutral zone. The neutral zone covers 1 cm from top to bottom of the Petri dish between the left and right sides. It thereby separates both halfs and covers the transition from pure agarose to agarose plus gustatory stimulus. By

subtracting the number of larvae on the pure agarose side (#nS) from the number of larvae on the stimulus side (#S) divided by the total number of counted larvae (#TOTAL), a preference index for the respective chemosensory stimulus was calculated:

PREF = (#S - #nS) /#TOTAL

Negative PREF values indicate avoidance, whereas positive PREF values represent attractiveness.

For olfactory preference tests, a similar assay was used except that olfactory stimuli were presented in custom-made Teflon containers with perforated lids presented on only pure agarose containing test plates. As olfactory stimuli amyl acetate (AM, Fluka Cat. No.: 46022; CAS No.: 628-63-7; diluted 1:250 in paraffin oil, Fluka Cat. No.: 76235, CAS No.: 8012-95-1), benzaldehyde (BA, Fluka Cat. No.: 12010, CAS No.: 100-52-7; undiluted), 1-heptanol (HEP, Sigma Aldrich Cat. No.: H2805, CAS No.: 111-70-6; undiluted) and 1-nonanol (NON, Sigma Aldrich Cat. No.: 131210, CAS No.: 143-08-8; undiluted) were used.
